# Camrelizumab (a PD-1 inhibitor) plus apatinib (an VEGFR-2 inhibitor) and hepatic artery infusion chemotherapy for hepatocellular carcinoma in Barcelona Clinic Liver Cancer stage C (TRIPLET): a phase II study

**DOI:** 10.1038/s41392-023-01663-6

**Published:** 2023-10-27

**Authors:** Tian-Qi Zhang, Zhi-Jun Geng, Meng-Xuan Zuo, Ji-Bin Li, Jin-Hua Huang, Zi-Lin Huang, Pei-Hong Wu, Yang-Kui Gu

**Affiliations:** 1 Department of Minimally Invasive Interventional Therapy, Sun Yat-sen University Cancer Center, Guangzhou, P. R. China; 2 State Key Laboratory of Oncology in South China, Collaborative Innovation Center for Cancer Medicine, Guangzhou, P. R. China; 3 Department of Radiology, Sun Yat-sen University Cancer Center, Guangzhou, P. R. China; 4 Department of Clinical Research, Sun Yat-sen University Cancer Center, Guangzhou, P. R. China

**Keywords:** Gastrointestinal cancer, Drug development

## Abstract

Hepatic arterial infusion chemotherapy (HAIC) using a combination of oxaliplatin, fluorouracil, and leucovorin (FOLFOX) has shown promise for hepatocellular carcinoma (HCC) patients classified under Barcelona Clinic Liver Cancer (BCLC) stage C. In China, the combined therapy of camrelizumab and apatinib is now an approved first-line approach for inoperable HCC. This study (NCT04191889) evaluated the benefit of combining camrelizumab and apatinib with HAIC-FOLFOX for HCC patients in BCLC stage C. Eligible patients were given a maximum of six cycles of HAIC-FOLFOX, along with camrelizumab and apatinib, until either disease progression or intolerable toxicities emerged. The primary outcome measured was the objective response rate (ORR) based on the Response Evaluation Criteria in Solid Tumors (RECIST) v1.1. Thirty-five patients were enrolled. Based on RECIST v1.1 criteria, the confirmed ORR stood at 77.1% (95% CI: 59.9% to 89.6%), with a disease control rate of 97.1% (95% CI: 85.1% to 99.9%). The median progression-free survival was 10.38 months (95% CI: 7.79 to 12.45). Patient quality of life had a transient deterioration within four cycles of treatment, and generally recovered thereafter. The most frequent grade ≥3 or above treatment-related adverse events included reduced lymphocyte count (37.1%) and diminished neutrophil count (34.3%). The combination of camrelizumab, apatinib, and HAIC demonstrated encouraging results and manageable safety concerns for HCC at BCLC stage C.

## Introduction

Worldwide, primary liver cancer ranks as the sixth most frequent cancer diagnosis and stands third in terms of cancer-related mortality, with hepatocellular carcinoma (HCC) accounting for 75–80% of these primary liver cancer cases.^1^ Over 80% of HCC instances in China originate from Hepatitis B virus (HBV) infections, and the 5-year rates of overall survival (OS) range between a mere 10% to 18%.^2–4^ HCC that has progressed to the Barcelona Clinic Liver Cancer (BCLC) stage C, which is marked by a performance status of 1–2, significant vascular invasion such as portal vein tumor thrombosis (PVTT), and/or had extrahepatic metastasis, typically excludes surgical interventions.^5^ Besides, the presence of tumor thrombus and extra-hepatic metastasis in HCC are risk factors for poor prognosis.^5^ Despite the recent increase in therapeutic options BCLC stage C patients, there remains a need for new treatment strategies.

The union of PD-(L)1 inhibitors with agents targeting VEGF has marked a pivotal shift in HCC management, as evidenced initially in the IMbrave150 study.^6^ When antiangiogenic drugs are paired with anti-PD-1 treatments, there’s a suppression of immune checkpoint activity and an enhancement in T-cell functionality, leading to a more potent antitumor response compared to anti-PD-1 treatment alone.^7,8^ A combination of camrelizumab, a PD-1 antagonist, with apatinib, a tyrosine kinase inhibitor (TKI) focusing on VEGFR-2, has shown notable antitumor effects in advanced HCC cases.^9^ Recently, the international randomized controlled CARES-310 trial demonstrated that camrelizumab plus apatinib conferred a survival advantage over sorafenib for HCC patients with unresectable HCC.^10^ These findings have led to the approval of camrelizumab plus apatinib in China as an first-line treatment approach for unresectable HCC.

Hepatic arterial infusion chemotherapy (HAIC) using a combination of oxaliplatin, fluorouracil, and leucovorin (FOLFOX) is effective in decreasing the intrahepatic tumor burden, as it allows for the targeted delivery of chemotherapy drugs to the arteries supplying the tumor.^11^ HAIC-FOLFOX yielded significantly prolonged OS with a better overall safety profile compared to transarterial chemoembolization (TACE) for large HCC.^12^ The combined use of HAIC-FOLFOX and sorafenib has shown improved survival rates in HCC patients, in contrast to using sorafenib alone.^13^ HAIC-FOLFOX plus lenvatinib and toripalimab (an anti-PD-1 antibody) were found to be feasible in treating advanced HCC patients with high-risk features in a recent single-arm trial.^14^

With all above mentioned, it is hypothesized that camrelizumab and apatinib in combination with HAIC may further improve outcomes. Consequently, this investigation was designed to evaluate the therapeutic benefit of camrelizumab and apatinib when used alongside HAIC-FOLFOX in HCC patients at BCLC stage C.

## Results

### Patients

**Fig. 1:**
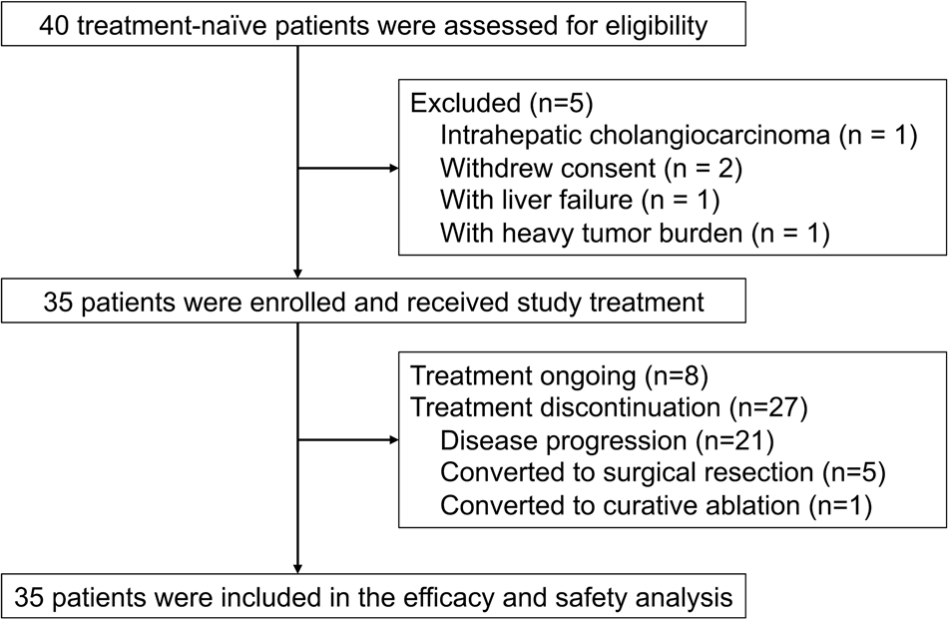
Patient flowchart

From April 13, 2020 to May 10, 2022, 35 eligible patients were enrolled (Fig. 1 and Supplementary Fig. S1). Most of the participants (91.4%) were male, with 42.9% being above the age of 50. All the cases originated from HBV infection. A total of 16 patients (45.7%) had a PVTT of Vp 3 or 4, and five patients (14.3%) developed extrahepatic metastasis prior to enrollment (Table 1).

**Table 1:** Baseline characteristics of patients

Variables	All patients (*n* = 35)
Age, years, median (range)	46 (27–67)
≥50	15 (42.9%)
<50	20 (57.1%)
Sex, *n* (%)	
Male	32 (91.4%)
Female	3 (8.6%)
BMI, kg/m^2^, median (range)	20.6 (17.3–28.8)
≥24	10 (28.6%)
<24	25 (71.4%)
Etiology, *n* (%)	
Hepatitis B	35 (100.00%)
ECOG performance status score, *n* (%)	
0	16 (45.7%)
1	18 (51.4%)
2	1 (2.9%)
Child-Pugh score, *n* (%)	
5	32 (91.4%)
6	3 (8.6%)
ALBI grade, *n* (%)	
Grade 1	29 (82.9%)
Grade 2	6 (17.1%)
AFP, ng/mL, *n* (%)	
≥400	19 (54.3%)
<400	16 (45.7%)
PIVKA-II, mAU/mL, median (range)	8392.4 (42.0–75000.0)
Tumor size, cm, median (range)	10.5 (4.7–18.9)
≥10	21 (60.0%)
<10	14 (40.0%)
Venous tumor thrombus, *n* (%)	31 (88.6%)
PVTT, *n* (%)	
Vp1	3 (8.6%)
Vp2	6 (17.1%)
Vp3	8 (22.9%)
Vp4	8 (22.9%)
Absent	10 (28.6%)
IVCTT, *n* (%)	
Hepatic vein invasion	8 (22.9%)
IVC invasion	2 (5.7%)
Absent	25 (71.4%)
Extrahepatic metastasis, *n* (%)	5 (14.3%)

*BMI* body mass index, *ECOG* Eastern Cooperative Oncology Group, *ALBI* albumin-bilirubin, *AFP* alpha-fetoprotein, *PIVKA-II* prothrombin in vitamin K absence II, *PVTT* portal vein tumor thrombosis, *IVCTT* inferior vena cava tumor thrombosis, *IVC* inferior vena cava

### Efficacy

Up to September 30, 2022, the median duration of follow-up stood at 23.10 months (95% confidence interval [CI], 17.44 to 28.76). The median cycles of HAIC were 6 (range, 4 to 6); the median cycles of camrelizumab were 9 (range, 4 to 32), and the median duration for apatinib was 9.1 months (range, 1.8 to 27.9).

**Fig. 2:**
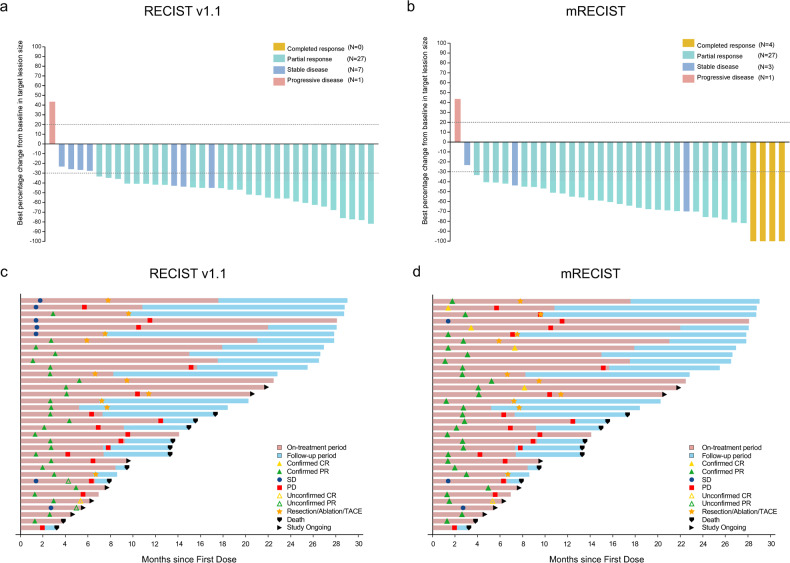
Treatment response and duration. **a** Best percentage changes from baseline in target lesions per RECIST v1.1; **b** best percentage changes from baseline in target lesions per mRECIST; **c** treatment exposure and response duration per RECIST v1.1; and **d** treatment exposure and response duration per mRECIST

**Fig. 3:**
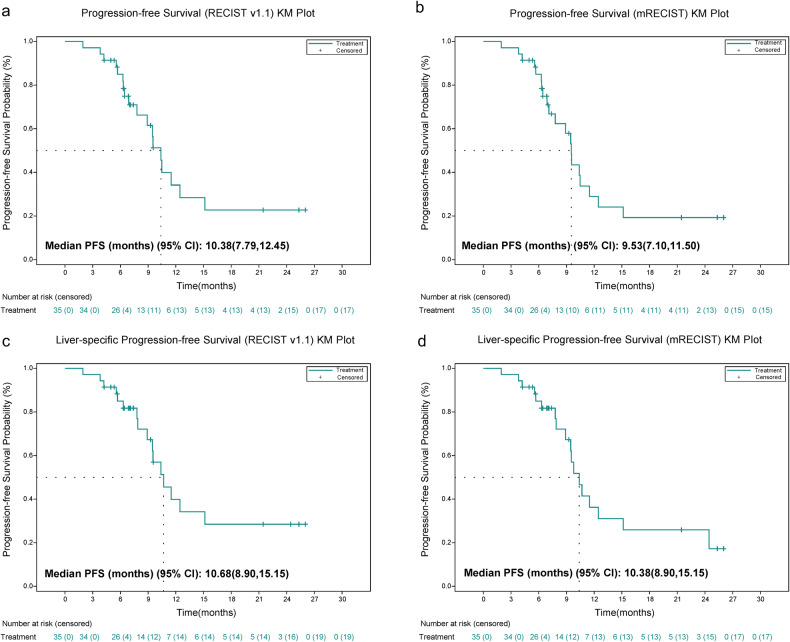
Survival analysis. **a** Kaplan–Meier curves of progression-free survival (PFS) per RECIST v1.1; **b** Kaplan–Meier curves of PFS per mRECIST; **c** Kaplan–Meier curves of liver-specific PFS per RECIST v1.1; **d** Kaplan–Meier curves of liver-specific PFS per mRECIST

Based on the Response Evaluation Criteria in Solid Tumors (RECIST) v1.1, the confirmed objective response rate (ORR) stood at 77.1% (95% CI: 59.9% to 89.6%), and the disease control rate (DCR) was 97.1% (95% CI: 85.1% to 99.9%) (Fig. 2). The confirmed ORR per modified RECIST (mRECIST) was 88.6% (95%CI, 73.3% to 96.8%). The subgroup analysis of ORR is presented in Supplementary Fig. S2. The ORR was consistent among all subgroups, including those with tumor size ≥10 cm or PVTT of Vp 3 or 4. The median time for progression-free survival (PFS) was established at 10.38 months (95% CI: 7.79 to 12.45), with the 6-, 12-, 18-month PFS rate of 85.0%, 34.2%, and 22.8%, respectively. When focusing on liver-specific PFS, the median duration was 10.68 months (95% CI: 8.90 to 15.15) (Fig. 3). The time to response (TTR) was 2.66 months (95% CI: 2.10 to 2.89), and the median duration of response (DoR) was 7.52 months (95% CI: 4.83 to 12.52). The median OS remains not reached, and the 6-, 12-, 24-month OS rates were 94.3%, 87.4%, and 65.0%, respectively (Table 2 and Supplementary Fig. S3). After the triple combination treatment, six of 35 patients (17.1%) achieved disease downstaging and received curative therapy (five patients underwent R0 resection, and one patient received curative ablation).

**Table 2:** Tumor response

Variables	All patients (*n* = 35)	
	RECIST v1.1 (*n* = 35)	mRECIST (*n* = 35)
Best objective response, *n* (%)		
Complete response	0	4 (11.4%)
Partial response	27 (77.1%)	27 (77.1%)
Stable disease	7 (20.0%)	3 (8.6%)
Progressive disease	1 (2.9%)	1 (2.9%)
Objective response rate, *n* (%)	27 (77.1%)	31 (88.6%)
95% CI	(59.9%, 89.6%)	(73.3%, 96.8%)
Disease control rate^a^, *n* (%)	34 (97.1%)	34 (97.1%)
95% CI	(85.1%, 99.9%)	(85.1%, 99.9%)
TTR, months, median (95% CI)	2.66 (2.10, 2.89)	2.63 (1.38, 2.73)
DOR, months, median (95% CI)	7.52 (4.83, 12.52)	6.70 (5.09, 9.66)
PFS, months, median (95% CI)	10.38 (7.79, 12.45)	9.53 (7.10, 11.50)
6-month PFS rate	85.0%	85.0%
12-month PFS rate	34.2%	28.9%
18-month PFS rate	22.8%	19.3%
Liver-specific PFS, months, median (95% CI)	10.68 (8.90, 15.15)	10.38 (8.90, 15.15)
6-month PFS rate	85.0%	85.0%
12-month PFS rate	39.9%	36.3%
18-month PFS rate	28.8%	25.9%

*TTR* time to response, *DOR* duration of response, *PFS* progression-free survival, *CI* confidence interval, *RECIST* Response Evaluation Criteria in Solid Tumors, *mRECIST* modified Response Evaluation Criteria in Solid Tumors

^a^For patients with stable disease, it should persist for a minimum of 6 weeks

### Exploratory endpoints

All patients were included in the quality of life (QoL) analysis. The global health status, as well as all functioning and most symptoms had a transient deterioration within four cycles of treatment, and generally recovered thereafter (Supplementary Fig. S4). The time to deterioration (TTD) was not reached (Supplementary Fig. S5). Following treatment, we observed a substantial reduction in the levels of prothrombin in vitamin K absence II and alpha-fetoprotein. During treatment, some patients exhibited a transient deterioration in liver function, as indicated by a temporary rise in the albumin-bilirubin (ALBI) score to level 2. However, following administration of liver-supportive care, the majority of these patients experienced a restoration or even improvement of liver function, with ALBI score returning to level 1 (Supplementary Fig. S6).

### Safety

All patients experienced treatment-related adverse events (TRAEs) (Table S1). TRAEs of grade ≥3 or above were evident in 26 patients (74.3%), with the predominant events being decreased lymphocyte count (37.1%) and reduced neutrophil count (34.3%) (Table 3). Reactive cutaneous capillary endothelial proliferation (RCCEP) was observed in 13 patients (37.1%), with the majority presenting at grade 1 (Table S2). Hypertension related to apatinib manifested in 14 (40.0%) patients, primarily at grades 1 and 2 (Table S3). HAIC-induced reductions in neutrophil and lymphocyte counts were observed in 29 (82.9%) and 27 (77.1%) patients, respectively (Table S4).

**Table 3:** Treatment-related adverse events of all grades occurring in more than 15% of patients

Events, *n* (%)	All patients (*n* = 35)		
	Any grade	Grade 1–2	Grade 3 or higher
Aspartate aminotransferase increased	34 (97.1)	24 (68.6)	10 (28.6)
Alanine aminotransferase increased	33 (94.3)	26 (74.3)	7 (20.0)
Hypoalbuminemia	31 (88.6)	31 (88.6)	0
Neutrophil count decreased	29 (82.9)	17 (48.6)	12 (34.3)
Lymphocyte count decreased	27 (77.1)	14 (40.0)	13 (37.1)
Anemia	25 (71.4)	23 (65.7)	2 (5.7)
Platelet count decreased	23 (65.7)	15 (42.9)	8 (22.9)
Blood bilirubin increased	22 (62.9)	19 (54.3)	3 (8.6)
Proteinuria	22 (62.9)	22 (62.9)	0
Abdominal pain	21 (60.0)	21 (60.0)	0
White blood cell decreased	20 (57.1)	14 (40.0)	6 (17.1)
Weight loss	17 (48.6)	17 (48.6)	0
Anorexia	17 (48.6)	17 (48.6)	0
Hyperglycemia	16 (45.7)	16 (45.7)	0
Hyponatremia	15 (42.9)	14 (40.0)	1 (2.9)
Hyperuricemia	15 (42.9)	15 (42.9)	0
Hypokalemia	14 (40.0)	12 (34.3)	2 (5.7)
Rash	14 (40.0)	12 (34.3)	2 (5.7)
Hypertension	14 (40.0)	9 (25.7)	5 (14.3)
Hand-foot syndrome	13 (37.1)	10 (28.6)	3 (8.6)
RCCEP	13 (37.1)	12 (34.3)	1 (2.9)
Diarrhea	13 (37.1)	12 (34.3)	1 (2.9)
Vomiting	12 (34.3)	12 (34.3)	0
Fatigue	11 (31.4)	11 (31.4)	0
Hematuria	11 (31.4)	11 (31.4)	0
Upper respiratory infection	9 (25.7)	9 (25.7)	0
Gingival hemorrhage	9 (25.7)	9 (25.7)	0
Fever	8 (22.9)	8 (22.9)	0
Oral mucositis	8 (22.9)	8 (22.9)	0
Gingivitis	8 (22.9)	8 (22.9)	0
Ascites	7 (20.0)	7 (20.0)	0
Epistaxis	7 (20.0)	7 (20.0)	0
Cough	6 (17.1)	6 (17.1)	0
Headache	6 (17.1)	6 (17.1)	0

*RCCEP* reactive cutaneous capillary endothelial proliferation

AEs prompted dose reductions in two patients, while four patients ceased treatment due to AEs (Table S1). A total of 13 patients experienced serious AE (SAE), with platelet count decreased (14.3%) as the most common (Table S5). Five (14.3%) patients experienced immune-related SAEs, including one case of immune dermatitis (grade 3), one case of RCCEP (grade 3), one case of thrombocytopenia (grade 3), and two cases of immune hepatitis (grade 3, grade 4, respectively); all of them recovered after camrelizumab discontinuation and steroid treatment.

## Discussion

The therapeutic landscape of HCC has undergone significant advancements with the integration of antiangiogenic agents and immunotherapy. As demonstrated in the IMbrave150 study, atezolizumab and bevacizumab achieved an ORR of 27.3% and a median PFS spanning 6.8 months for HCC.^6^ Likewise, the CARES-301 study showed that camrelizumab plus apatinib yielded an ORR of 25.4% and a median PFS of 5.6 months.^10^ The triple combination treatment in our study showed a numerically higher ORR (77.1% per RECIST v1.1) and prolonged PFS (10.38 months) than that of above studies, which suggested that HCC in BCLC stage C may benefit from the addition of HAIC to camrelizumab and apatinib. A synergistic antitumor effect of HAIC-FOLFOX, apatinib, and camrelizumab may be responsible for survival benefit in our study.^7,15,16^ In HAIC, chemotherapy agents are infused directly into tumors for about 50 h. The oxaliplatin and fluorouracil in HAIC-FOLFOX induces tumor cell death, which release tumor antigens.^17^ Apatinib is started on day 8 after HAIC treatment to allow for a recovery period, which is beneficial for the transient liver function damage caused by HAIC, and to reduce the liver toxicity of combined chemotherapy and other drugs. Additionally, low-dose apatinib induces prolonged vascular normalization, which reduces tumor hypoxia and acidosis and enhanced the efficacy of the infiltrating immune cells.^18^ Anti-PD-1 therapy targets immune checkpoint and activates cytotoxic T lymphocyte function, thereby providing a more favorable antitumor activity.^7,8^ Therefore, camrelizumab is initiated in the second cycle of treatment.

The prognosis of HCC patients with high-risk features remains suboptimal. Our study also enrolled patients at high-risk, including eight patients with Vp 4 PVTT and 21 with tumors larger than 10 cm. According to the subgroup analysis, the combination of camrelizumab, apatinib and HAIC-FOLFOX yielded high response rate for these patients. Notably, one patient who initially met the inclusion criteria with an ECOG PS score of 1 experienced a deterioration to 2 before treatment. Despite this, the patient showed improvement in ECOG PS score and achieved a PR after treatment. This observation suggests that the regimen may extend benefits to patients with poorer ECOG PS scores, although further research is needed to confirm this. Consistent with our results, Lai et al. also demonstrated that combining HAIC-FOLFOX with lenvatinib and toripalimab is a viable option for HCC patients exhibiting high-risk characteristics.^14^ Taking these results into account, camrelizumab, apatinib and HAIC was beneficial for patients with unresectable HCC, even for those at high risk.

After the triple combination treatment, six patients (17.1%) achieved disease downstaging and received curative therapy, including five patients who underwent R0 resection, and one patient who received curative ablation. This advantage has also been discussed in several other studies about HAIC. According to a study in patients with large HCC, a notably increased rate of curative surgical resection was evident in the HAIC-FOLFOX group compared to the TACE group (24% vs. 12%).^12^ Furthermore, disease downstaging was achieved in up to 12% of HCC patients who underwent treatment with either HAIC in combination with sorafenib or HAIC as a standalone therapy.^13,19^

The QoL of patients with malignancies are of paramount importance, particularly since it is proven to affect the long-term prognosis of the patients.^20^ In our analysis, compared with the baseline, most patients experienced a transient deterioration in QoL within four cycles of treatment, followed by a gradual improvement. Interestingly, it appeared that the improvement of QoL coincided with the control of disease. The REFLECT study also found that responders were related to a lower risk of deterioration and better scores in QoL than non-responders.^21^ This emphasized the importance of effective tumor control to improve the QoL of patients.

In our findings, the combination of HAIC with camrelizumab and apatinib was generally well-tolerated. The frequency and severity of AEs encountered in our cohort aligned with the established safety profiles of HAIC and the combination of camrelizumab and apatinib, as indicated in prior research.^9,22^ The combination of agents did not appear to induce any unusual overlapping toxicity. The occurrence of grade 3 TRAEs was consistent with the results from the RESCUE study.^9^ What is noteworthy is that we made some modifications in HAIC by reducing the dose oxaliplatin from 130 mg/m^2^ to 85 mg/m^2^, and administering an analgesic agent concurrently with the infusion to avoid substantial abdominal pain caused by direct injection of oxaliplatin into the intrahepatic artery.

This trial is not without its limitations. First, the lack of a control arm in our single-arm design makes it difficult to definitively attribute the observed benefits solely to the addition of systemic therapy following HAIC. Second, the study’s sample size was limited, and although we met the predetermined criteria of Simon’s two-stage design, early termination of the study could potentially result in an overestimation of the ORR. To comprehensively evaluate the merits of the triple combination, we have initiated a randomized controlled phase 3 trial (NCT05313282) aimed at assessing its benefit compared to camrelizumab plus apatinib in HCC patients with BCLC Stage C. Third, the study exclusively enrolled patients with HBV infection, representing 85–90% of HCC cases in China.^23,24^ While this focus aligns with the prevalent etiological factors of HCC in China, it may restrict the extrapolation of our findings to HCC cases stemming from other causes. It’s important to highlight that China accounts for half of both the global incidence and mortality of HCC.^1,25^ Thus, the study’s focus on HBV-related HCC holds significant implications not just for China, but for global HCC treatment strategies as well. Forth, the average age of the patients was 46, which may appear young but is actually reflective of the typical HCC patient demographic in China. The mean age of HCC diagnosis in China is 52, significantly younger than that in Western countries and Japan.^26,27^ This variation can largely be ascribed to the chronic HBV infection, which is a primary factor leading to HCC in China. In our cohort, 57.1% of the patients were under the age of 50, and 42.9% were 50 or older, mirroring the age distribution for HBV-related HCC in China.

In summary, the regimen combining camrelizumab, apatinib, and HAIC demonstrated efficacy and safety in treating BCLC stage C HCC patients. Conclusions with more powerful evidence may be obtained from the phase 3 study in the near future.

## Methods

### Patients

TRIPLET represents a single-arm trial. Essential criteria for patient inclusion encompassed: age between 18 and 70 years; a clinical or pathological HCC diagnosis based on the American Association for the Study of Liver Diseases criteria;^28^ classification within BCLC stage C;^5^ no prior anti-tumor treatment exposure; presence of at least one measurable intrahepatic tumor as per RECIST v1.1; an Eastern Cooperative Oncology Group (ECOG) performance status score of either 0 or 1; and a Child-Pugh score of ≤7. Patients with autoimmune disease, uncontrolled hypertension or high risk of bleeding were ruled out. Detailed eligibility criteria can be found in protocol.

### Ethics statements

All participants gave their written consent prior to being included in the study. The study protocol was approved by the Institutional Review Boards (B2019-187-01). This research has been registered on ClinicalTrials.gov under the identifier NCT04191889.

### Procedures

After enrollment, patient underwent up to six cycles of HAIC, each lasting 21 days, with the FOLFOX regimen. This regimen consisted of a 2-h infusion of oxaliplatin at 85 mg/m^2^, a 2–3-h administration of leucovorin at 400 mg/m^2^, and a 46-h delivery of fluorouracil at 2500 mg/m^2^. In addition, all participants were administered camrelizumab (200 mg intravenously, commencing on day 4 of the second HAIC cycle and repeated every 21 days) and apatinib (250 mg daily, taken orally, beginning on day 8 of the initial HAIC cycle) until disease progression or unacceptable toxicities (Supplementary Fig. S1).

HAIC was performed by inserting a 5-French Yashiro catheter (Terumo Corporation, Tokyo, Japan) through the femoral artery with a 2.7-French microcatheter inside, and then, advancing the tip of the microcatheter to the tumor-feeding artery, guided by concurrent arteriography. Tumors were accessed via the right or left hepatic artery. When a tumor demonstrated additional blood supply from extrahepatic sources, catheter tip was positioned in the main feeding artery. Besides, branch arteries were embolized with blank microspheres. In the event that there is a short access path from intrahepatic arteries leading to chemical agents flowing into the gastroduodenal artery, coils would be used to embolize it. The administration of chemotherapeutic agents for HAIC was completed within 3 days after hepatic catheter placement. The catheter and sheath were removed after the completion of each HAIC.

Combination therapy was discontinued when disease progression, disease downstaging to have an opportunity to perform curative treatment, unacceptable toxicities, or death occurred. In the event of grade ≥3 or serious TRAEs, the related study treatment should be discontinued, and the other two were allowed to continue.

Every 6 weeks until treatment completion, tumor responses were evaluated using dynamic contrast-enhanced computed tomography (CT) or magnetic resonance imaging (MRI), following both RECIST v1.1 and mRECIST criteria. In the event that a patient achieves complete response (CR) or partial response (PR), the response must be confirmed no less than 4 weeks of the initial evaluation. AEs during the treatment were recorded or graded according to the National Cancer Institute Common Terminology Criteria for Adverse Events (NCI-CTCAE) 4.0.

### Outcomes

The primary objective was to determine the ORR according to RECIST v1.1, which was defined as the percentage of participants experiencing either a CR or PR. Secondary outcomes encompassed ORR as determined by mRECIST, DCR, TTR, DoR, PFS, liver-specific PFS, OS, along with 6-month and 12-month PFS and OS rates. A comprehensive definition of these secondary endpoints is provided in the study protocol. An exploratory objective of this study was to evaluate the QoL, gauged using the European Organization for Research and Treatment of Cancer QoL questionnaire (EORTC QLQ-C30) [30]. This assessment was conducted at the study’s onset and then every 6 weeks until the treatment concluded. Scores from the EORTC QLQ-C30 were converted to a scale ranging from 0 to 100. On this scale, a higher score indicates improved functioning but increased symptom severity. The TTD in QoL was determined as treatment initiation to the first observed decline of 10 or more points from the baseline score.

### Statistical analysis

A Simon’s 2-stage design was adopted in this study, with a one-sided *α* of 2.5% and to guarantee the power over 80%. The null hypothesis of ORR per RECIST v1.1 was 40%, and the alternative hypothesis was 60.8%. If a response is observed in over 11 out of the initial 26 evaluated patients during the first phase, an additional 21 patients would be recruited for the study. The treatment would be deemed worthy of further investigation if more than 25 patients exhibit a response.

All participants who underwent at least one study treatment were considered for both efficacy and safety analyses. Both ORR and DCR were presented with their two-sided 95% CI: calculated using the Clopper-Pearson approach. The median values for time-to-event variables were determined through the Kaplan–Meier technique, and their respective 95% CI were derived using the Brookmeyer and Crowley method. All statistical evaluations were carried out using SAS® software (version 9.4, SAS Institute Inc, Cary, USA).

### Supplementary information

Supplementary material

## Data Availability

The datasets supporting the conclusions of this manuscript are available in the Sun Yat-sen University Cancer Center Research Data Deposit repository (RDDA2020001690).
