# Supplementary material for: Camrelizumab (a PD-1 inhibitor) plus apatinib (an VEGFR-2 inhibitor) and hepatic artery infusion chemotherapy for hepatocellular carcinoma in Barcelona Clinic Liver Cancer stage C (TRIPLET): a phase II study
Source: Signal Transduct Target Ther. 2023 Oct 27;8:413. doi: 10.1038/s41392-023-01663-6 (PMC10603153; doi:10.1038/s41392-023-01663-6)
Supplement: Supplementary file 1 — Supplementary material [file 41392_2023_1663_MOESM1_ESM.docx]

Supplementary Materials for

**Camrelizumab (a PD-1 inhibitor) plus apatinib (an VEGFR-2 inhibitor) and hepatic artery infusion chemotherapy for hepatocellular carcinoma in Barcelona Clinic Liver Cancer stage C (TRIPLET): a phase II study**

Tian-Qi Zhang, Zhi-Jun Geng, Meng-Xuan Zuo, Ji-Bin Li, Jin-Hua Huang, Zi-Lin Huang, Pei-Hong Wu, Yang-Kui Gu

Correspondence to: Yang-Kui Gu ([guyk@sysucc.org.cn](mailto:guyk@sysucc.org.cn)), Pei-Hong Wu ([wuph@sysucc.org.cn](mailto:wuph@sysucc.org.cn))

**This PDF file includes:**

Figures S1 to S6

Tables S1 to S5

Data S1

Figure S1. Study design. HCC: hepatocellular carcinoma; AASLD, American Association for the Study of Liver Diseases; BCLC, Barcelona Clinic Liver Cancer; ECOG, Eastern Cooperative Oncology Group; HAIC, Hepatic Artery Infusion Chemotherapy; ORR, objective response rate.

Figure S2. Subgroup analysis of objective response rate A) per RECIST 1v.1; B) per mRECIST.

Figure S3. Kaplan-Meier curve of overall survival.

Figure S4. Health-related quality of life over time. A) global health status; B) functioning scores; C) and D) symptom scores. Data are presented as mean and standard error (SEM)

Figure S5. Time to deterioration of quality of life. A) global health status; B) functioning scores; C) and D) symptom scores.

Figure S6. Changes in biomarkers over time. A) change in alpha-fetoprotein (AFP) from baseline; B) change in prothrombin in vitamin K absence II (PIVKA-II) from baseline; C) change in albumin-bilirubin (ALBI) over time. The best of response (partial response, stable disease or progressive disease) was assessed based on RECIST v1.1 criteria.

Table S1. Summary of adverse events

| Events, n (%) | All patients (n=35) | |
| --- | --- | --- |
|  | Any grade | Grade 3 or higher |
| SAE | 13 (37.1) | 13 (37.1) |
| Treatment-related SAE | 13 (37.1) | 13 (37.1) |
| Camrelizumab-related SAE | 11 (31.4) | 11 (31.4) |
| Apatinib-related SAE | 9 (25.7) | 8 (22.9) |
| HAIC-related SAE | 8 (22.9) | 8 (22.9) |
| Immune-related AE | 18 (51.4) | 6 (17.1) |
| TEAE leading to dose reduction | 2 (5.7) | 0 |
| TEAE leading to camrelizumab dose reduction | 0 | 0 |
| TEAE leading to apatinib dose reduction | 2 (5.7) | 0 |
| TEAE leading to HAIC related chemotherapy dose reduction | 0 | 0 |
| TEAE leading to dose interruption | 19 (54.3) | 10 (28.6) |
| TEAE leading to camrelizumab interruption | 4 (11.4) | 3 (8.6) |
| TEAE leading to apatinib interruption | 19 (54.3) | 9 (25.7) |
| TEAE leading to HAIC interruption | 2 (5.7) | 1 (2.9) |
| TEAE leading to treatment discontinuation | 4 (11.4) | 4 (11.4) |
| TEAE leading to camrelizumab discontinuation | 2 (5.7) | 2 (5.7) |
| TEAE leading to apatinib discontinuation | 3 (8.6) | 3 (8.6) |
| TEAE leading to HAIC discontinuation | 0 | 0 |

TEAE: treatment-emergent adverse event; HAIC: hepatic arterial infusion chemotherapy; SAE: serious adverse event.

Table S2. Camrelizumab-related adverse events occurring in more than 15% of patients

| Events, n (%) | All patients (n=35) | | |
| --- | --- | --- | --- |
|  | Any grade | Grade 1-2 | Grade 3 or higher |
| Aspartate aminotransferase increased | 34 (97.1) | 24 (68.6) | 10 (28.6) |
| Alanine aminotransferase increased | 33 (94.3) | 26 (74.3) | 7 (20.0) |
| Platelet count decreased | 23 (65.7) | 15 (42.9) | 8 (22.9) |
| Blood bilirubin increased | 22 (62.9) | 19 (54.3) | 3 (8.6) |
| Hyperglycemia | 16 (45.7) | 16 (45.7) | 0 |
| Rash | 14 (40.0) | 12 (34.3) | 2 (5.7) |
| RCCEP | 13 (37.1) | 12 (34.3) | 1 (2.9) |
| Gingival hemorrhage | 9 (25.7) | 9 (25.7) | 0 |
| Hypertension | 8 (22.9) | 8 (22.9) | 0 |
| Fever | 8 (22.9) | 8 (22.9) | 0 |
| Oral mucositis | 8 (22.9) | 8 (22.9) | 0 |
| ﻿Gingivitis | 8 (22.9) | 8 (22.9) | 0 |

RCCEP: reactive cutaneous capillary endothelial proliferation.

Table S3. Apatinib-related adverse events occurring in more than 15% of patients

| Events, n (%) | All patients (n=35) | | |
| --- | --- | --- | --- |
|  | Any grade | Grade 1-2 | Grade 3 or higher |
| Aspartate aminotransferase increased | 34 (97.1) | 24 (68.6) | 10 (28.6) |
| Alanine aminotransferase increased | 33 (94.3) | 26 (74.3) | 7 (20.0) |
| Hypoalbuminemia | 31 (88.6) | 31 (88.6) | 0 |
| Anemia | 25 (71.4) | 23 (65.7) | 2 (5.7) |
| Blood bilirubin increased | 22 (62.9) | 19 (54.3) | 3 (8.6) |
| Proteinuria | 22 (62.9) | 22 (62.9) | 0 |
| Abdominal pain | 21 (60.0) | 21 (60.0) | 0 |
| White blood cell decreased | 20 (57.1) | 14 (40.0) | 6 (17.1) |
| Weight loss | 17 (48.6) | 17 (48.6) | 0 |
| Anorexia | 17 (48.6) | 17 (48.6) | 0 |
| Hyponatremia | 15 (42.9) | 14 (40.0) | 1 (2.9) |
| Hyperuricemia | 15 (42.9) | 15 (42.9) | 0 |
| Hypokalemia | 14 (40.0) | 12 (34.3) | 2 (5.7) |
| Rash | 14 (40.0) | 12 (34.3) | 2 (5.7) |
| Hypertension | 14 (40.0) | 9 (25.7) | 5 (14.3) |
| Hand-foot syndrome | 13 (37.1) | 10 (28.6) | 3 (8.6) |
| Diarrhea | 13 (37.1) | 12 (34.3) | 1 (2.9) |
| Fatigue | 11 (31.4) | 11 (31.4) | 0 |
| Hematuria | 11 (31.4) | 11 (31.4) | 0 |
| Upper respiratory infection | 9 (25.7) | 9 (25.7) | 0 |
| Fever | 8 (22.9) | 8 (22.9) | 0 |
| Oral mucositis | 8 (22.9) | 8 (22.9) | 0 |
| Ascites | 7 (20.0) | 7 (20.0) | 0 |
| Epistaxis | 7 (20.0) | 7 (20.0) | 0 |
| Headache | 6 (17.1) | 6 (17.1) | 0 |

Table S4. Hepatic arterial infusion chemotherapy-related adverse events occurring in more than 15% of patients

| Events, n (%) | All patients (n=35) | | |
| --- | --- | --- | --- |
|  | Any grade | Grade 1-2 | Grade 3 or higher |
| Aspartate aminotransferase increased | 34 (97.1) | 24 (68.6) | 10 (28.6) |
| Alanine aminotransferase increased | 33 (94.3) | 26 (74.3) | 7 (20.0) |
| Hypoalbuminemia | 31 (88.6) | 31 (88.6) | 0 |
| Neutrophil count decreased | 29 (82.9) | 17 (48.6) | 12 (34.3) |
| Lymphocyte count decreased | 27 (77.1) | 14 (40.0) | 13 (37.1) |
| Anemia | 25 (71.4) | 23 (65.7) | 2 (5.7) |
| Platelet count decreased | 23 (65.7) | 15 (42.9) | 8 (22.9) |
| Blood bilirubin increased | 22 (62.9) | 19 (54.3) | 3 (8.6) |
| Abdominal pain | 21 (60.0) | 21 (60.0) | 0 |
| White blood cell decreased | 20 (57.1) | 14 (40.0) | 6 (17.1) |
| Weight loss | 17 (48.6) | 17 (48.6) | 0 |
| Anorexia | 17 (48.6) | 17 (48.6) | 0 |
| Hyponatremia | 15 (42.9) | 14 (40.0) | 1 (2.9) |
| Diarrhea | 13 (37.1) | 12 (34.3) | 1 (2.9) |
| Vomiting | 12 (34.3) | 12 (34.3) | 0 |
| Fatigue | 11 (31.4) | 11 (31.4) | 0 |
| Upper respiratory infection | 9 (25.7) | 9 (25.7) | 0 |
| ﻿Fever | 8 (22.9) | 8 (22.9) | 0 |
| Ascites | 7 (20.0) | 7 (20.0) | 0 |
| Cough | 6 (17.1) | 6 (17.1) | 0 |
| Headache | 6 (17.1) | 6 (17.1) | 0 |

Table S5. Serious adverse events

| Events, n (%) | All patients (n=35) | |
| --- | --- | --- |
|  | Any grade | Grade 3 or higher |
| Platelet count decreased | 5 (14.3) | 5 (14.3) |
| Autoimmune hepatitis | 2 (5.7) | 1 (2.9) |
| Blood bilirubin increased | 2 (5.7) | 2 (5.7) |
| Hand-foot syndrome | 2 (5.7) | 2 (5.7) |
| Gastrointestinal hemorrhage | 2 (5.7) | 0 |
| Dermatitis | 1 (2.9) | 1 (2.9) |
| RCCEP | 1 (2.9) | 1 (2.9) |
| White blood cell decreased | 1 (2.9) | 1 (2.9) |
| Rash | 1 (2.9) | 1 (2.9) |
| Aspartate aminotransferase increased | 1 (2.9) | 1 (2.9) |
| Alanine aminotransferase increased | 1 (2.9) | 1 (2.9) |
| Intracranial hemorrhage | 1 (2.9) | 1 (2.9) |
| Hypertension | 1 (2.9) | 1 (2.9) |

RCCEP: reactive cutaneous capillary endothelial proliferation.

Data S1

**Camrelizumab (a PD-1 inhibitor) plus apatinib (an VEGFR-2 inhibitor) and hepatic artery infusion chemotherapy for hepatocellular carcinoma in Barcelona Clinic Liver Cancer stage C (TRIPLET): a phase II study**

**Protocol**

Content

[Abbreviations and Terms 5](#_Toc112598133)

[1. STUDY BACKGROUND 8](#_Toc112598134)

[1.1 Overview of Hepatocellular Carcinoma 8](#_Toc112598135)

[1.2 Advances in Breakthroughs Continue 8](#_Toc112598136)

[1.3 Advances in Immunotherapy 10](#_Toc112598137)

[1.4 Advanced in Arterial Infusion Chemotherapy 12](#_Toc112598138)

[1.5 Combination of Any Two of Targeted Therapy, Immunization and Infusion Is Superior to Monotherapy 13](#_Toc112598139)

[1.5.1 Advantages of Combination of Arterial Infusion Chemotherapy and Targeted Therapy 13](#_Toc112598140)

[1.5.2 The Combined Effect of Arterial Infusion Chemotherapy and Immunotherapy Is Better 13](#_Toc112598141)

[1.5.3 Research Progress of Targeted Therapy + Immunotherapy for Advanced Liver Cancer 14](#_Toc112598142)

[1.6 Research of HAIC Combined With Antiangiogenic Targeted Therapy and Immunology in the Era of Immunotherapy 17](#_Toc112598143)

[1.7 Study Basis 17](#_Toc112598144)

[1.8 Potential Risks and Benefits 18](#_Toc112598145)

[1.8.1 Known Potential Risks 18](#_Toc112598146)

[1.8.2 Known Possible Benefits 19](#_Toc112598147)

[2. STUDY OBJECTIVES 20](#_Toc112598148)

[2.1 Primary Objective 20](#_Toc112598149)

[2.2 Secondary Objectives 20](#_Toc112598150)

[3. STUDY DESIDN 20](#_Toc112598151)

[3.1 Overall Design 20](#_Toc112598152)

[3.2 Medication Regimen Design Basis 21](#_Toc112598153)

[3.3 Sample Size Estimation 22](#_Toc112598154)

[3.4 Ways to Reduce Bias 22](#_Toc112598155)

[3.4.1 Enrollment/Randomization/Blinding Steps 22](#_Toc112598156)

[3.4.2 Blind Evaluation 22](#_Toc112598157)

[3.4.3 Break the Blind 22](#_Toc112598158)

[4. SUBJECT SELECTION AND WITHDRAWAL 23](#_Toc112598159)

[4.1 Inclusion Criteria 23](#_Toc112598160)

[4.2 Exclusion Criteria 23](#_Toc112598161)

[4.3 Subjects Withdraw from Study or Discontinue Study Treatment 25](#_Toc112598162)

[4.3.1 Criteria for Discontinuing Treatment 25](#_Toc112598163)

[4.3.2 Steps to Withdraw From the Study or Discontinue Study Treatment 26](#_Toc112598164)

[4.4 Study Termination Criteria 26](#_Toc112598165)

[4.5 End-of-trial Definition 26](#_Toc112598166)

[5. STUDY DRUGS 26](#_Toc112598167)

[5.1 Overview of Study Drugs 27](#_Toc112598168)

[5.1.1The Way to Get Drugs 27](#_Toc112598169)

[5.1.2 Dosage Form, Appearance, Packaging and Label of Drugs 27](#_Toc112598170)

[5.1.3. Preservation and Stability of Drugs 28](#_Toc112598171)

[5.1.4 Preparations of Drugs 28](#_Toc112598172)

[5.1.5 Usage of Drugs 29](#_Toc112598173)

[5.1.6 Special Dosing Equipment Considerations 29](#_Toc112598174)

[5.2 Treatment Plan 29](#_Toc112598175)

[5.3 Dose Adjustment 30](#_Toc112598176)

[5.4 Management, Distribution and Recovery of Drugs 36](#_Toc112598177)

[5.5 Concomitant Therapy 36](#_Toc112598178)

[5.5.1 Other Anti-tumor/Anti-cancer or study drugs 36](#_Toc112598179)

[5.5.2 Supportive Care 37](#_Toc112598180)

[5.5.3 Immune Preparation 37](#_Toc112598181)

[5.5.4 Drugs That May Interact with Apatinib 37](#_Toc112598182)

[6.2 Treatment Visit 38](#_Toc112598183)

[6.3 End of Treatment/Withdrawal 42](#_Toc112598184)

[6.4 Follow-up 43](#_Toc112598185)

[7. STUDY ASSESSMENT 43](#_Toc112598186)

[7.1 Evaluation of Effectiveness 43](#_Toc112598187)

[7.1.1 Effectiveness Indicator 43](#_Toc112598188)

[7.1.2 Effectiveness Evaluation Criteria 45](#_Toc112598189)

[7.1.3 Tumor Lesions Evaluations 46](#_Toc112598190)

[7.2 Safety Analyses 47](#_Toc112598191)

[7.2.1Baseline Signs and Symptoms 47](#_Toc112598192)

[7.2.2 Physical Examination and Vital Signs 47](#_Toc112598193)

[7.2.3 Laboratory Safety Assessment 47](#_Toc112598194)

[7.2.4 Vital Signs and Physical Examination and Weight Measurement 47](#_Toc112598195)

[7.3 Biomarker Analyses 47](#_Toc112598196)

[8. ADVERSE EVENT REPORTING 48](#_Toc112598197)

[8.1 Definition of Adverse Event (AE) 48](#_Toc112598198)

[8.2 Assessment of Adverse Events 48](#_Toc112598199)

[8.3 Recording of Adverse Events 49](#_Toc112598200)

[8.4 Evaluation of the Correlation Between Adverse Events and Drugs 49](#_Toc112598201)

[8.5 Serious Adverse Event (SAE) 49](#_Toc112598202)

[8.6 Management of Serious Adverse Events 50](#_Toc112598203)

[9. ADVICE ON HANDLING ADVERSE EVENTS 50](#_Toc112598204)

[9.1 Immune-related Adverse Event 50](#_Toc112598205)

[9.2 Principles of Handling Immune-related Adverse Events 50](#_Toc112598206)

[9.3 Infusion Reactions 52](#_Toc112598207)

[9.4 Symptomatic Management of Apatinib-related Adverse Reactions 55](#_Toc112598208)

[10.STATIATICAL ANALYSES 57](#_Toc112598209)

[10.1 Determination of Sample Size 57](#_Toc112598210)

[10.2 Intention to Treat Population 58](#_Toc112598211)

[10.3 Endpoint Analysis and Statistical Methods 58](#_Toc112598214)

[10.3.1 General Methods of Statistical Analysis 58](#_Toc112598217)

[10.3.2 Efficacy Analyses 58](#_Toc112598218)

[10.3.2.1 Primary Endpoint Analysis 58](#_Toc112598219)

[10.3.2.2 Secondary Endpoint Analyses 59](#_Toc112598220)

[10.3.3 Safety Analysis 60](#_Toc112598221)

[10.3.3.1 Exposure of Medication 60](#_Toc112598222)

[10.3.3.2 Adverse Events 60](#_Toc112598223)

[10.3.4 Baseline Characteristics of Subjects 60](#_Toc112598224)

[10.3.5 Exploratory Analysis 61](#_Toc112598225)

[11.DATA COLLECTION AND MANAGEMENT 62](#_Toc112598226)

[11.1 Case Report Form 62](#_Toc112598227)

[11.2 Data Management 62](#_Toc112598228)

[12.Ethics 62](#_Toc112598229)

[12.1 Responsibility of the Investigator 63](#_Toc112598230)

[12.2 Ethics Committee 63](#_Toc112598231)

[12.3 Information of Subjects 63](#_Toc112598232)

[12.4 Informed Consent 63](#_Toc112598233)

[REFERENCES: 65](#_Toc112598234)

# Abbreviations and Terms

| Abbreviation and Terms | Full name |
| --- | --- |
| ADA | Anti-drug antibody |
| ADRs | Adverse drug reactions |
| AE | Adverse event |
| AFP | A-fetoprotein |
| AKP | Alkaline phosphatase |
| ALT | Alanine aminotransferase |
| ANC | Absolute neutrophil count |
| APTT | Activeated partial thromboplastin time |
| ASCO | American Society of Clinical Oncology |
| AST | Aspartate aminotransferase |
| AUC | Area under curve |
| BP | Blood pressure |
| BUN | Blood urea nitrogen |
| B超 | B-type ultrasound |
| Ca | Calcium |
| CFDA | China Food and Drug Administration |
| Cl | Chlorine |
| Cr | Creatinine |
| CR | Complete response |
| CRF | Case report form |
| CT | Computed tomography |
| CTCAE | Common Terminology Criteria for Adverse Events |
| DBIL | Direct bilirubin |
| DCR | Disease control rate |
| DoR | During of response |
| DLT | Dose limited toxicity |
| ECG | Electrocardiogram |
| ECOG score | Eastern Cooperative Oncology Group |
| eCRF | Electronic Case report form |
| EDC | Electroinc data collection |
| FAS | Full analyse set |
| FIB | Fibrinogen |
| FDA | Food and Drug Administration |
| FT3 | free triiodothyronine |
| FT4 | free thyroxine |
| GCP | Good Clinical Practice |
| Glu | Glucose |
| Hb | Hemoglobin |
| HBV | Hepatitis B virus |
| HBsAg | Hepatitis B surface anitgen |
| HCV | Hepatitis C virus |
| irAE | Immune-related adverse event |
| INR | International Normalized Ratio |
| IRB | Institutional review board |
| ITT | intend to treat |
| K | Potassium |
| LLN | lower limits of normal |
| mPFS | median progression-free survival |
| MRI | Magnetic Resonance Imaging |
| MTD | Maximum torlerate dose |
| Na | Sodium |
| NCCN | National Comprehensive Cancer Network |
| NCI-CTC | National cancer institute Common Terminology Criteria |
| ORR | Objective response rate |
| OS | Overall survival |
| P | Phosphorus |
| PD | Progressive disease |
| PD-1/PD-L1 | Programmed death 1/programmed death  ligand 1 |
| PFS | Progression-Free-Survival |
| PK | Pharmacokinetics |
| PLT | Platelet |
| PPS | Per-Protocol Set |
| PR | Partial response |
| PT | Prothrombin time |
| QoL | Quality of life |
| RBC | Red blood cell |
| RO | Receptor occupancy |
| RECIST | Response evaluation criteria in solid tumors |
| SAE | Serious adverse event |
| SAS | SAS Programme |
| SD | Stable disease |
| SOP | Standard Operation Procedure |
| SS | Safety Analysis set |
| TB | Total bilirubin |
| TC | Total cholesterol |
| TCM | Traditional Chinese Medicine |
| TG | Triglyceride |
| TP | Plasma total protein |
| TMB | Tumor mutant burden |
| TSH | Thyroid-stimulating hormone |
| TTR | Time to response |
| UA | Blood uric acid |
| UICC | Union for International Cancer Control |
| ULN | Upper limit of normal |
| WBC | White blood cell |

# 1. STUDY BACKGROUND

## 1.1 Overview of Hepatocellular Carcinoma

Primary liver cancer is a common malignant tumor of the digestive system worldwide, with about 854,000 new cases and 810,000 deaths each year. There is a high incidence of liver cancer in our country, with about 466,000 new cases and 422,000 deaths from liver cancer every year [1]. In primary liver cancer, hepatocellular carcinoma (HCC) is the main pathological type, accounting for about 90%. In the subclinical phase, patients are often asymptomatic or subclinical. At the time of diagnosis, most patients have reached topically advanced stage or developed distant metastasis, and the natural survival time is only 3 to 4 months. Surgery is the first choice for treatment (liver resection and liver transplantation), but only less than 30% of patients are suitable for surgery, and some patients can use non-surgical topical treatments including ablation, interventional therapy, radiotherapy etc. [2] Although patients can use surgical and non-surgical topical treatment, the recurrence rate is a little high. For patients without surgical and non-surgical indications and for patients with recurrence and metastasis after treatment, multiple guidelines worldwide recommend the use of palliative system (systemic) treatment [3].

Sorafenib, a multi-targeted kinase inhibitor, is the first systemic drug approved for the treatment of unresectable and distant metastases in advanced HCC [4]. However, in practical clinical applications, the efficacy of sorafenib in the treatment of advanced liver cancer is not ideal, especially for liver cancer with tumor thrombus in the trunk and branches of portal vein, the median survival time of patients is only 3 to 6 months. HCC in our country is highly heterogeneous in terms of etiology, eipdemiological characteristics, molecular biological behavior, clinical manifestations and staging, treatment strategies and prognosis etc., which are different from those in Europe, America and Japan. Therefore, HCC treatment in our country cannot be completely copied from the experience of Europe, America and Japan. It’s necessary to obtain high-level evidence-based medical evidence based on the Chinese population, so as to formulate a comprehensive HCC diagnosis and treatment standard to guide clinical practice.

## 1.2 Advances in Breakthroughs Continue

In the past two years, on the basis of sorafenib, several targeted drugs for first- and second-line treatment of advanced liver cancer have been successively approved. Lenvatinib was approved for the first-line treatment of advance liver cancer through a head-to-head non-inferiority REFLECT study with sorafenib [5]. Regorafenib improved TTP (3.2 vs 1.0 months), ORR (11% vs 4%) and DCR (65% vs 36%), and prolonged survival (10.6 vs 7.8 months) in patients with advanced HCC who failed in sorafenib therapy, so it was approved for the second-line treatment of liver cancer [6].

Apatinib mesylate is used for patients who have progressed or recurred advanced gastric adenocarcinoma or gastroesophageal junction adenocarcinoma after receiving at least 2 previous systemic chemotherapy. The recommended dosage regimen is 850mg, orally(PO), once daily(QD), in 28-day cycle (excluding the dosing suspension time), and it is continuously used until disease progresses or intolerable adverse reactions occur. If grade 3/4 hematological or non-hematological adverse reactions occur, it is recommended to suspend the drug (not more than 2 weeks), or reduce the dose to 750mg or 500mg.

Primary hepatocellular carcinoma (HCC) is also a typical hypervascular tumor, and its occurrence, development, metastasis and invasion are closely related to angiogenesis. From 2010 to 2013, the 81st Hospital of the Chinese People’s Liberation Army led the completion of a randomized, open-label, multi-center phase Ⅱ clinical study of apatinib mesylate tablets in the treatment of advanced hepatocellular carcinoma, with a total of 121 subjects taking medication. In the full analysis set, the median time to progression (mTTP) and median overall survival (mOS) were respectively 4.21 months and 9.71 months in the 850mg group, and respectively 3.32 months and 9.82 months in the 750mg group. The differences in mTTP and mOS between the 850mg and the 750mg groups were not statistically significant. The incidence of adverse reactions in the 850 mg groups was respectively 95.71% and 90.20%, and the incidence of severe adverse reactions were respectively 58.57% and 58.82%, and the differences were not statistically significant. Therefore, a well-tolerated 750mg was recommended as the dosage for phase Ⅲ clinical studies. The recommended dosage regimen was 750mg, PO, QD, in 28-day cycle (excluding the dosing suspension time), and it was continuously used until the disease progressed or intolerable adverse reactions occurred. If grade 3/4 hematological or non-hematological adverse reactions occurred, it was recommended to suspend the drug (not more than 2 weeks), or reduce the dose to 500mg or 250mg.

Apatinib is currently undergoing a randomized (2:1), open-label, placebo-controlled, multi-center phase Ⅲ clinical trial to observe the efficacy and safety of apatinib in the second-line treatment of advanced HCC. Advanced HCC patients with sorefenib or systemic chemotherapy failure, recurrence, or side effect intolerance were recruited. As of December 2017, 400 subjects had been enrolled. Adverse events that had been observed include proteinuria, increased blood pressure, thrombocytopenia, hand-foot syndrome, and increased total bilirubin etc. For more information on apatinib mesylate tablets, please refer to the drug insert of apatinib mesylate tablets and the investigator’s brochure provided by the sponsor.

Based on the large-scale randomized controlled clinical study data above, the maximum tolerated dose of apatinib mesylate monotherapy is 850mg QD, and the minimum effective dose is 250mg QD. The main adverse reactions are hand-foot skin reaction, hypertension, elevated transaminase, elevated bilirubin, leukopenia, thrombocytopenia, diarrhea, esophagitis, nausea, fatigue, etc., most of which were mild to moderate reactions. Considering that the combined toxicity and efficacy are likely to be doubled, 250mg QD is selected as the combined dose in this trial, and the dosing frequency of apatinib can be adjusted according to the degree of tolerance of adverse reactions.

## 1.3 Advances in Immunotherapy

Sorafenib opened the era of HCC targeted therapy in 2007, and Nivolumab opened the era of HCC immunotherapy ten years later. The liver itself is an important immune organ. It recognizes, activates and removes pathogens in a timely and effective manner through a variety of immune cells, and at the same time, through precise balance regulation, it prevents excessive inflammatory responses from causing damage to the body. In the process of maintaining the homeostasis of liver, hepatic sinusoidal endothelial cells, hepatic macrophages, dendritic cells and hepatic innate lymphocytes play important immunoregulatory functions [10-11].

However, in the state of tumor burden, this balance is disturbed. Persistent viral infection and tumor neoantigen stimulation lead to the formation of an immunosuppressive state in the liver, which promotes the formation and progression of liver cancer [12]. Cell populations with immunosuppressive functions, such as regulatory T cells (Treg) and myeloid suppressor cells (MDSC), are significantly increased in liver cancer tissues [13-16]. Continued exposure to antigen also overexpresses CTLA4, PD1, and LAG3 co-suppressive signaling molecules on the surface of tumor-specific lymphocytes, ultimately rendering T cells in a state of incapacitation without an immune response [17-18].

Therefore, targeting at the immunosuppressive mechanism in liver cancer and reversing the disabled state of T cells to reactivate T cells and exert anti-tumor effects is an important direction of liver cancer immunotherapy. In recent years, significant progress has been made in cancer treatment. Small-molecule drug-targeted therapies that inhibit tumor angiogenesis and intrinsic drivers of cancer cell growth, and immunotherapies that enhance patients’ anti-tumor immunity have been approved by regulatory agencies in various countries. With the deepening understanding of the body’s immune system and the rapid development of biotechnology, immunotherapy has become an important means of tumor treatment, and occupies an increasingly important position in the comprehensive tumor treatment system.

Targeted therapy can elicit significant clinical responses in a variety of tumor types, but these responses are short-lived and prone to tumor immune escape and clinical relapse in the months following the initial response. By contrast, immunotherapy can also be applied to a variety of tumors and can produce relatively durable responses in some patients. Unlike conventional chemotherapy and targeted therapy that directly act on tumor cells, immunotherapy can directly kill tumor cells on the one hand, and more importantly, by acting on the immune system, enhance the body’s immune response, and ultimately prolong the patient’s survival.

The full name of PD-1 is Programmeddeath-1, which is a negative co-stimulatory molecule discovered in recent years. PD-L1 and PD-L2 are ligands of PD-1 and can specifically bind to PD-1. Through the high expression of PD-L1 molecule, tumor cells combine with PD-1 molecules on T lymphocytes to transmit negative regulatory signals, leading to the induction of apoptosis and immune incompetence of tumor antigen-specific T cells, allowing tumor cells to escape the body’s immune surveillance and killing. PD-1 inhibitors are a new class of tumor immunotherapy drugs that have attracted much attention, which regulate the anti-tumor activity of T lymphocytes by blocking the PD-1/PD-L1 signaling pathway, and cause tumor apoptosis.

Since 2014, the U.S. Food and Drug Administration (FDA) has successively approved anti-PD-1 monoclonal antibodies based on breakthrough efficacy. Bristol-Myers Squibb’s Nivolumab and Merck’s Pembrolizumab for the treatment of advanced melanoma and non-small cell lung cancer, renal cell carcinoma, head and neck cancer, Hodgkin’s lymphoma, and hepatocellular carcinoma (HCC) patients who have received sorafenib treatment, etc., including patients with advanced stage, standard treatment failure, and no effective treatment methods, are also promoted to first-line and second-line treatment. In addition, the anti-PD-1 monoclonal antibody has a long-lasting efficacy and relatively mild adverse reactions so a series of hundreds of clinical trials have been conducted internationally for advanced solid tumors and malignant hematological diseases, including monotherapy and combination therapy, whose preliminary trial results demonstrated higher response rates and long-term survival rates compared to current therapies.

On September 23th, 2017, the FDA approved Nivolumab for patients with hepatocellular carcinoma (HCC) after sorafenib treatment based on the data of Checkmate-040 trail, marking the official entry of the immunotherapy era for liver cancer treatment. This was a phase Ⅰ/Ⅱ dose escalation and expansion clinical trial of single-arm treatment for advanced hepatocellular carcinoma evaluating Nivolumab effectiveness and safety: 262 enrolled patients (48 in the dose-escalation phase and 214 in the dose-expansion phase).

In the dose-escalation study, patients received Nivolumab every 2 weeks, 0.1 to 10mg/kg. In the dose-expansion study, they received Nivolumab every 2 weeks, 3mg/kg. The primary endpoints of the dose-escalation phase were safety and tolerability, and the primary endpoint of the dose-expansion phase was ORR. The results showed that Nivolumab had a manageable safety profile and acceptable tolerability. The ORR was 15% in the dose-escalation phase and 20% in the dose-expansion phase. It was important to emphasize that the trial results showed that Nivolumab remained effective in HBV-positive patients. The 12-month survival rate of first-line Nivolumab in patients who did not use sorafenib was 73%, and the 12-month overall survival rate in patients who used sorafenib was 58% to 60%.

Despite the unprecedented success of this type of therapy, it cannot be ignored that a large proportion of the patient population still does not respond to this therapy. From the current point of view, combination therapy will be one of the inevitable trends in the future development of tumor immunotherapy. How to carry out combination therapy is a problem that needs to be further solved in the medical field.

Currently, there are a large number of ongoing phase Ⅰ/Ⅱ clinical trials combining targeted therapy and immunotherapy. The rationale in support of these combination therapies is that the two therapies combine different immunological and tumor biological mechanisms that enhance anti-tumor activity. In addition, there is some evidence that therapy can enhance certain links in the “cancer-immunity cycle” (such as tumor antigenicity, T cell activation/transportation/infiltration, etc.), which synergistically enhances immunotherapy. In particular, targeted therapy for the mitogen-activated protein kinase (MAPK) pathway and the vascular endothelial growth factor (VEGF) pathway, representative drugs including sunitinib, can not only directly affect tumor cell growth and tumor angiogenesis, but also affects tumor cell antigenicity and intratumoral T-cell infiltration, which affects the patient’s immune response beyond their role in tumor biology, providing a powerful combination therapy basis for combination therapy.

Combination therapies currently carried out mainly focus on treatment of advanced melanoma, lung cancer, and renal cell carcinoma. In addition, they also show great clinical prospects in the treatment fields of liver cancer, ovarian cancer, colorectal cancer, head and neck cancer, and Hodgkin’s lymphoma etc. Some key factors should be considered in the clinical development of combination therapy, such as optimizing the dosing regimen, minimizing treatment-related toxicity, selecting an appropriate endpoint to evaluate efficacy, etc.

This clinical study will involve recombinant humanized anti-PD-1 monoclonal antibody injection (camrelizumab), a new class Ⅰ drug for therapeutic biological products developed by Jiangsu Hengrui Medicine Co., Ltd. The preclinical research data shows that camrelizumab has comparable in vivo efficacy and safety compared with similar foreign drugs. Since 2015, Hengrui has simultaneously carried out phase Ⅰ/Ⅱ clinical trials of multiple tumor types in Australia and China, preliminarily verifying the safety, tolerability and efficacy of camrelizumab in the treatment of advanced solid tumors. Detailed information on camrelizumab is provided in the camrelizumab investigator brochure provided by the sponsor.

## 1.4 Advances in Arterial Infusion Chemotherapy

The treatment principle of hepatic arterial infusion chemotherapy (HAIC) is to continuously infuse chemotherapeutic drugs through the hepatic artery. Compared with systemic chemotherapy, it can achieve a higher concentration of chemotherapeutic drugs in the local area of liver cancer, reduce systemic side effects and enhance the killing effect on tumor cells. Compared with hepatic arterial chemoembolization (TACE), it can also reduce the liver function damage caused by arterial embolization, and at the same time, infusion chemotherapy drugs are used to replace the short-term bolus injection of chemotherapy drugs in TACE, which can make the chemotherapy drugs act on liver cancer tumor cells for a longer time and enhance the efficacy of chemotherapy. A number of studies have reported that the FOLFOX regimen HAIC has definite curative effect, good safety and efficacy in the treatment of liver cancer [21]. Zhao et al. used HAIC for the treatment of advanced liver cancer, and its ORR was up to 28.6%, and they found that compared with sorafenib, both OS and PFS were significantly prolonged (OS 14.5 mos vs. 7.0 mos; PFS 7.1 mos vs. 3.3 mos) [22].

## 1.5 Advantages of Combination of Any Two of Targeted Therapy, Immunization and Infusion

### 1.5.1 Advantages of Combination of Arterial Infusion Chemotherapy and Targeted Therapy

In May 2019, Professor Shi Ming’s team reported a blockbuster study result in JAMA Oncology based on the results of the phase Ⅱ study with satisfactory early efficacy, suggesting that HAIC combined with systemic targeted therapy had better efficacy. Compared with sorafenib monotherapy, the combination of sorafenib and HAIC could prolong OS from 7.13 months to 13.37 months, and PFS from 2.6 months to 7.03 months. Moreover, the sorafenib combined with HAIC group also had a higher disease remission rate (40.8% vs. 2.46%). Although the toxicity of combination therapy was slightly higher than that of sorafenib monotherapy, these adverse reactions were tolerable [23].

### 1.5.2 The Combined Effect of Arterial Infusion Chemotherapy and Immunotherapy Is Better

A review published in Cancer Immunol Res in 2015 reported that various chemotherapeutic drugs, including cyclophosphamide, paclitaxel, cisplatin, and temozolomide, can be used at low doses to modulate tumor immunity in a time-dependent manner. Among them, the adjuvant activity of cyclophosphamide was the most studied. A single low-dose cyclophosphamide administered 1-3 days prior to antigen exposure overcomes systemic immune tolerance to enhance antibody and T cell responses, while the same treatment given after or concurrently with antigen exposure induces antigen-specific tolerance. In preclinical models, low-dose cyclophosphamide given 1 day before immunotherapy depleted Tregs, promoted DC maturation, shifted the CD4+ T helper phenotype from type 2 to type 1, induced Th17 differentiation, and promoted evolution by IFNα-secreting a sustained CD44hi T cellular memory response. Clinically, a cyclophosphamide dose of 200-300mg/m^2^ given 1 day before vaccination or 600mg/m^2^ given 7 days before vaccination can reduce Tregs and metronomic cyclophosphamide was equally effective. In addition, low-dose doxorubicin administered 7 days after vaccination can enhance vaccine activity and delay tumor growth. Combining cyclophosphamide and doxorubicin at this low dose and schedule had the greatest effect, curing some mice. In this model, cyclophosphamide selectively depleted regulatory T cells, allowing the specific recruitment of high-affinity tumor-specific T cells in animals cured of their tumors (not in mice with tumors growing) [24].

The success of immune checkpoint antagonists (PD-1) heralds a new era in cancer therapy, and harnessing the power of the immune system to treat cancer is becoming a key strategy in clinical management. Understanding the cellular and molecular mechanisms by which traditional cancer therapies interact, and their dose- and time-dependent activities, is critical for effectively integrating different immunotherapies (including immune checkpoint antagonists and cancer vaccine) with chemotherapy and targeted molecular therapies treatment. Chemotherapeutic drugs have the effect of enhancing immunotherapy at different stages in the tumor immune cycle. In the case of insignificant single drug effect, combined use with PD-1 inhibitor may improve the effect of tumor immune response. Currently, a phase Ⅲ study of camrelizumab plus FOLFOX4 regimen in the first-line treatment of advanced HCC is also recruiting patients. This study selects patients with histologically confirmed advanced HCC, the primary endpoint of which is OS, and the secondary endpoint are ORR, TTP, DCR, DOR, PFS, 9- and 12-month OS rates, safety, immunogenicity and pharmacokinetics of the camrelizumab, hoping to obtain satisfactory efficacy [25-26].

### 1.5.3 Research Progress of Targeted Therapy + Immunotherapy for Advanced Liver Cancer

In recent years, both persistent tumor angiogenesis and immune escape have been included in the ten characteristics of tumors. Tumor cells can secrete a large number of immunosuppressive factors, inhibit the function of immune cells, and recruit a large number of immunosuppressive cells into tumor tissues. These immunosuppressive cells can secrete immunosuppressive factors, and then build a positive feedback immunosuppressive network. Finally, these molecules and cells together form a functionally complex tumor immunosuppressive micro-environment, making the tumor focus an “immune privilege zone” where antigen-specific T cells cannot function.

At 2017 Nature review reported the results of three preclinical studies showing that PD-1/PD-L1 blockade can sensitize tumors to anti-angiogenic therapy and improve their efficacy, and vice versa. The combination of anti-angiogenesis and immunotherapy can produce “a whole greater than the sum of the parts”, which has attracted widespread attention. Tumors can inhibit immune function in various ways: 1.VEGFR expressed by tumor cells can inhibit the maturation of DC cells, induce the expression of PD-L1, up-regulate Treg cells and inhibit the differentiation of hematopoietic stem cells, thereby causing immunosuppression; 2.Due to the ①structural changes, ②permeability changes, ③abnormal vascular perfusion and other characteristics of tumor neovascularization, the abnormal vasculature of the tumor will cause immunosuppression in the tumor micro-environment; leading to hypoxia, increased lactic acid and possible necrosis of tumor tissue, thereby activating immunosuppressive and inhibiting Teff. At the same time, a variety of immunosuppressive factors in the tumor micro-environment lead to dysfunction of immune cells or restrict their entry into the tumor [27-28].

However, anti-VEGF drugs can sever abnormal vascular tissue and promote the normalized growth of blood vessels, resulting in the reduction of inhibitory Treg regulatory cells in the tumor micro-environment and the improvement of intratumoral oxygen supply and pH levels. At the same time, a large number of T cells can be delivered to the tumor tissue, promoting tumor immunoediting [29].

Studies have shown that both immunotherapy and anti-angiogenic targeted therapy act on the tumor micro-environment (TEM), and there is a complex interaction between tumor immune micro-environment reprogramming and tumor vascular remodeling. Anti-angiogenic drugs that inhibit VEGF and its receptor (VEGFR) signal transduction pathway positively enhance the immune effect. It is currently believed that the mechanism of the synergistic effect of immune combined with anti-angiogenic therapy may include three aspects: (1)Both VEGF antibodies and VEGFR-targeted TKIs anti-angiogenic drugs can reduce the number of myeloid-derived suppressor cells (MDSCs) and regulatory T cells (Tregs) in the tumor micro-environment, and reduce the activity of these immunosuppressive cells, remodeling the tumor micro-environment; (2)VEGF can induce the expression of PD-L1 in mature dendritic cells (DCs) and affect the function of DCs to present antigens, while anti-angiogenic drugs can promote the more effective initiation and activation of T cells by blocking VEGF-mediated inhibition of DCs maturation and enhancing the function of DCs antigen presentation; (3)Anti-angiogenic drugs can normalize tumor vascular structure, induce the secretion of adhension molecules on the luminal surface of endothelial cells of tumor blood vessels, promote the infiltration of immune cells into tumor tissue (cell recruitment), improve TEM, and finally relieve immunosuppression.

Tumor immune checkpoint therapy is a current research hotspot, and a series of studies on immunotherapy combined with anti-angiogenic therapy are being carried out normally. Hodi et al. published a phase I clinical study of bevacizumab combined with Ipilimumab monoclonal antibody in the treatment of unresectable stage Ⅲ/Ⅳ malignant melanoma in Cancer Immunol Res in 2014. The dosing regimen was Ipilimumab 3 or 10mg/kg every 3 weeks for four doses followed by maintenance every 12 weeks and bevacizumab 7.5 or 15mg/kg every 3 weeks once. A total of 46 patients with metastatic malignant melanoma were included in the study. The most common adverse reactions included fatigue (35 patients), rash (32 patients), headache (25 patients) and cold (23 patients). High-grade adverse reactions occurred in 13 patients, dose-limiting toxicity (DLT) occurred in 2 patients, and there were no treatment-related deaths. PR was observed in 8 patients, DCR was 67.4%, and median OS was 25.1 months.

The IMpower150 was a multi-center, open-label, randomized, controlled phase Ⅲ study which evaluated chemotherapy combined with anti-angiogenic therapy in combination with the PD-L1 inhibitor Atezolizumab for first-line treatment of advanced non-squamous NSCLC. A total of 1202 patients with newly diagnosed advanced NSCLC were enrolled in this study and randomly divided into three groups: group A: paclitaxel+carboplatin+Atezolizumab; group B: group A combined with bevacizumab; group C: conventional treatment mode (paclitaxel+carboplatin+bevacizumab). In the 2018 ASCO annual meeting, the study reported that in the ITT-WT population, the median OS of the 4-drug combination regimen group was 19.2 months, the risk of death was reduced by 22%, and the median OS was 4.5 months longer than the control group; a separate analysis of 13% of EGFR/ALK-positive patients in this study found that the 4-drug combination regimen had a very significant OS benefit, with a 46% reduction in the overall risk of death. The IMpower150 study was the first phase Ⅲ clinical study of immune combined with anti-angiogenic therapy combined with chemotherapy, and has reached the endpoint of the study. It has clinically confirmed that immune combined with anti-angiogenic therapy has a synergistic effect, which provides an important revelation and confidence for subsequent similar studies.

It has been confirmed by animal experiments that apatinib and camrelizumab can regulate the tumor micro-environment by inhibiting VEGFR2 and PD-L1, and effectively inhibit tumors.

In addition, in a phase Ⅰ clinical study of camrelizumab combined with apatinib in the treatment of advanced liver, gastric or gastroesophageal junction cancer, PD-1 200mg Q2W plus apatinib 250mg QD was first determined by dose escalation. A dose-expansion study was then conducted with endpoints of ORR, OS, and safety. The results showed that among the 16 patients with liver cancer whose efficacy could be evaluated, the ORR was 50.0%, and the DCR was 93.8%. When the dose of apatinib was 250mg, the median PFS was 7.2 months, and the median OS had not yet been reached. 7 of the 8 patients with PR continued to be treated, and 5 of them were treated for more than 49 weeks. Camrelizumab combined with apatinib can effectively reduce the incidence of adverse reactions. Treatment-related adverse reactions (all grades) occurred in ≥10% of patients, and no treatment-related adverse reactions resulted in death. There was no RCEP in the apatinib 125mg and 500mg groups, and only 4 cases in the apatinib 250mg group. The combined anti-VEGF drug doubled the efficacy of the single drug, and the objective response rate, disease control rate and PFS were significantly increased, and at the same time the incidence of RCEP was reduced to less than 10%. At present, a randomized, controlled, open-label, international multi-center phase Ⅲ clinical study of camrelizumab combined with apatinib versus sorafenib in the first-line treatment of advanced HCC has been approved by the US FDA and it is the first PD-1 inhibitor in China to carry out an international multi-center phase Ⅲ clinical trial [25].

Therefore, low-dose anti-angiogenic drugs can inhibit tumor angiogenesis on the one hand, but also reduce immunosuppression by inducing normalization of blood vessels, enhance effector immune cell infiltration, and enhance the effect of anti-tumor immunity.

## 1.6 Study of HAIC Combined With Antiangiogenic Targeted Therapy and Immunology in the Era of Immunotherapy

To sum up, for unresectable advanced liver cancer, HAIC, anti-angiogenesis targeted therapy, and immunotherapy each have their own important positions, and the combination of any two of them can produce “a whole greater than the sum of the parts”. So, can the combination of the three treatment methods further improve the efficacy of advanced liver cancer?

At present, there is no clinical study on HAIC combined with immune checkpoint inhibitors and small molecule anti-angiogenic targeted drugs in the treatment of hepatocellular carcinoma. Therefore, we plan to conduct a prospective phase Ⅱ clinical study to preliminarily explore the safety and efficacy of the combination of HAIC and camrelizumab and apatinib mesylate in the treatment of advanced hepatocellular carcinoma, in order to accumulate experience for further research and provide new treatment concepts and methods for the treatment of advanced liver cancer.

## 1.7 Study Basis

In the study of camrelizumab combined with apatinib in the treatment of advanced hepatocellular carcinoma by Prof. Xu Jianming, Prof. Xu’s team observed that camrelizumab 200mg fixed dose combined with apatinib 250 mg, QD orally, was a tolerable dose. As of December 25th, 2017, a total of 14 patients with advanced hepatocellular carcinoma were enrolled in the study for camrelizumab, 200mg fixed dose combined with apatinib 125mg, and apatnib 250mg, QD orally dose level. Among them, a total of 4 subjects were enrolled in the study for camrelizumab, 200mg fixed dose combined with apatinib 125mg, QD orally dose level, and 2 subjects were escalated to 250mg apatinib during the treatment period, QD. A total of 10 subjects were enrolled in the study for camrelizumab, 200mg fixed dose combined with apatinib 250mg, QD orally dose level. The enrolled subjects were all HBV positive, and 11 of the 14 subjects failed sorafenib treatment. A total of 13 subjects completed at least one efficacy evaluation after enrollment and a total of 7 cases pf partial remission (PR) and 5 cases of stable disease (SD) were observed. The 6-week disease control rate was over 90%, and most subjects continued to benefit from the treatment with a median duration of more than 7 months.

In addition to the efficacy exploration of camrelizumab combined with apatinib in advanced hepatocellular carcinoma, tolerance observation and preliminary efficacy exploration in non-squamous and non-small cell lung cancer were also conducted. Preliminary results showed that camrelizumab combined with apatinib was also safe and tolerable in the lung cancer, and the combination had a more significant advantage in efficacy than PD-1 antibody alone.

A number of studies have confirmed the safety and tolerability of camrelizumab combined with apatinib, and the preliminary efficacy suggests that the combination is better than the single-agent PD-1 antibody.

In May 2019, the study results reported by Professor Shi Ming’s team in JAMA Oncology suggested that HAIC combined with systemic targeted therapy had better efficacy. Although the toxicity of combination therapy was slightly higher than that of sorafenib monotherapy, these adverse effects were tolerable.

Based on these published research results, we design this study to explore whether HAIC combined with targeted immune drugs can further improve clinical efficacy of advanced liver cancer.

## 1.8 Potential Risks and Benefits

### 1.8.1 Known Potential Risks

Any drug or therapy under investigation may have unforeseen or even serious side effects. A skin toxicity event hemangioma (RCCEP, not yet reported in other anti-PD-1 IgG4 antibody studies) was known to be reported in 60.5% of subjects treated with camrelizumab monotherapy. Among subjects with hemangioma, 6 subjects underwent surgical removal of the hemangioma and/or hospitalization for prolonged clinical observation. Therefore, hemangioma events in these subjects were classified as SAEs. All reported hemangioma degrees were ≤ grade 2. Disappearance of the hemangioma was observed after camrelizumab was discontinued. However, camrelizumab combined with apatinib therapy could reduce the incidence of cutaneous capillary hemangioma (RCCEP) to 9.5%.

In clinical studies of camrelizumab conducted in Australia and China, 7 subjects (1.1%) reported pneumonia SAE and 3 subjects (0.5%) reported interstitial lung diseases SAE.

Other immune-related AEs frequently reported in other anti-PD-1 antibodies, considered class effects, were also observed in clinical studies of camrelizumab, which included elevated AST (73 patients, 11.7%), elevated ALT (58 patients, 9.3%), rash (57 patients, 9.1%; preferred terms include rash, macular rash, maculopaular rash, erythematous eruption, impetigo, and itchy rash), diarrhea (44 cases, 7.1%), hypothyroidism (37 cases, 5.9%), and hyperthyroidism (8 cases, 1.3%). These events were considered likely to be related to the study drug due to their high incidence and the consistency with the safety profile of other anti-PD-1 antibodies. Notably, only one diarrhea SAE was reported, and no colitis SAE occurred. Therefore, many diarrheal events may not represent immune-related AEs, but rather the underlying condition. In addition, many patients with elevated AST and elevated ALT were liver cancer patients. Nearly a quarter of the 659 subjects enrolled in the Chinese camrelizumab study were patients with hepatocellular carcinoma.

The analysis showed that investigator-assessed immune-mediated adverse event with camrelizumab could not be ruled out primarily as skin toxicities (such as rash and skin hemangiomas). Most immune-mediated adverse events were grade I-Ⅱ.

The data above were compared with the adverse reactions reported by Bristol-Myers Squibb’s Nivolumab and Merck’s Pembrolizumab, which were similar anti-PD-1 antibodies on the market and the incidence and severity were low, and the overall adverse reaction profile was expected to be similar to Nicolumab and Pembrolizumab.

The study has developed recommendations for aggressive management of common side effects and a protocol-mandated dose adjustment plan to allow subjects to continue camrelizumab treatment on the premise of clinical benefit.

For all anti-PD-1 antibodies, the overall adverse reaction profile of monotherapy is better than that of tradition chemotherapy and targeted therapy. Compared with chemotherapy, the overall adverse reactions after combined chemotherapy are mainly manifested as fatigue, nausea, rash, hair loss and other adverse reactions, but the tolerability is good. Still need to pay special attention to immune-related adverse reactions, mainly including immune-related interstitial pneumonia, rash, thyroiditis, and low incidence (≤1%) vitiligo, colitis, nephritis, hepatitis, uveitis, adrenal insufficiency, nerve palsy, etc. Most of these immune-related adverse reactions are mild and controllable, and a few are serious adverse events that may even be life-threatening. The vast majority of immune-related adverse events can be adequately controlled under established toxicity protocols.

For all macromolecular protein monoclonal antibody drugs, other risks also include infusion reactions: mainly chill, shiver, cyanosis of the face and extremities followed by fever, which can be accompanied by nausea, vomiting, headache, dizziness, restlessness, delirium, etc., severe cases may have coma, decreased blood pressure, shock and respiratory failure and other symptoms, which are caused by curious factors such as pyrogen, drugs, impurities, low temperature of the liquid medicine, too high concentration of the liquid medicine and too fast infusion speed during intravenous infusion. As a fully humanized monoclonal antibody, camrelizumab has been reported to have a low incidence and mild degree of infusion reactions.

There may also be risks to subjects associated with medical examinations during the course of the study. More frequent imaging examinations will expose subjects to low-dose radiation etc. more frequently, but subjects with advanced liver cancer usually progress rapidly, and certain frequency of imaging examinations is also an essential step to determine whether the subject’s disease has progressed.

### 1.8.2 Known Possible Benefits

For BCLC stage C hepatocellular carcinoma, the standard treatment regimen is the targeted drug sorafenib or lenvatinib, and the domestically produced apatinib is applying for first-line treatment indications for liver cancer. However, only less than 10% of patients can observe tumor remission from first-line targeted drug treatment, and the cost of targeted drugs for approved indications is high, and there are a larger number of patients who cannot afford the treatment. More and more studies have shown that targeted combination immunotherapy (Lenvatinib+Keytruda, Avastin+Atezolizumab, Apatinib+Camrelizumab) produce “a whole greater than the sum of the parts” in advanced liver cancer, which is expected to change the first-line treatment guidelines for advanced liver cancer. HAIC combined with targeted drugs also show better clinical efficacy than single targeted drugs and the toxicity is tolerable. Our preliminary pre-experiments using HAIC combined with PD-1 monoclonal antibody and apatinib have achieved satisfactory preliminary data in the treatment of advanced hepatocellular carcinoma, and no serious adverse reactions occurred in any case. Therefore, participating in this study and receiving study drug treatment may lead to better clinical outcomes for patients with advanced hepatocellular carcinoma than the standard treatment recommended by current guidelines, providing these patients with a better treatment option. In addition, the immune drugs are fully funded by the company, and the financial burden of the enrolled patients is significantly reduced.

# 2. STUDY OBJECTIVES

## 2.1 Primary Objective

To evaluate the objective response rate (ORR) of HAIC combined with camrelizumab and apatinib in the treatment of BCLC stage C hepatocellular carcinoma.

## 2.2 Secondary Objectives

To evaluate the time to response (TTR) and duration of response (DOR) of HAIC combined with camrelizumab and apatinib in the treatment of BCLC stage C hepatocellular carcinoma;

To evaluate the disease control rate (DCR) of HAIC combined with camrelizumab and apatinib in the treatment of BCLC stage C hepatocellular carcinoma;

To evaluate the 6- and 12-month progression-free survival (PFS) and overall survival (OS) of HAIC combined with camrelizumab and apatinib in the treatment of BCLC stage C hepatocellular carcinoma;

To evaluate the median progression-free survival (mPFS), liver-specific PFS and overall survival (OS) of HAIC combined with camrelizumab and apatinib in the treatment of BCLC stage C hepatocellular carcinoma;

To evaluate the patient reported outcome (PRO) of HAIC combined with camrelizumab and apatinib;

To evaluate the safety and tolerability of HAIC combined with camrelizumab and apatinib.

# 3. STUDY DESIDN

## 3.1 Overall Design

The study is a prospective, single-arm, open-label phase Ⅱ clinical trial to evaluate the efficacy and safety of HAIC combined with camrelizumab and apatinib in the treatment of BCLC stage C hepatocellular carcinoma.

After fully informed and signing informed consent, the subjects who are qualified after screening will receive the simplified regimen of mFOLFOX7-HAIC+apatinib 250mg, QD orally plus camrelizumab 200mg, i.v., Q3W, with a 3-week treatment cycle, until a protocol-specific treatment discontinuation events occur. Subjects will continue to undergo post-treatment safety and survival follow-up after treatment. For subjects who end treatment for non-disease progression/death reasons, tumor progression follow-up will also be conducted after treatment.

After subjects are enrolled in the study, a safety visit will be conducted before the administration of camrelizumab on D_1_ of each treatment cycle. Imaging examinations are performed every 2 cycles to evaluate the efficacy until the end of treatment, withdrawal or death of informed consent. The study will also target subjects who sign up for biomarker sample collection and will also conduct biomarker blood and tumor sample collection at baseline and during the trial, including possible tumor biopsies at baseline.

## 3.2 Medication Regimen Design Basis

Several previous studies on a variety of tumors, including non-squamous, non-small cell lung cancer, hepatocellular carcinoma, intrahepatic cholangiocarcinoma and gastric adenocarcinoma have been reported in literature, which have confirmed: camrelizumab 200mg fixed dose combined with apatinib 250mg, QD orally is a tolerable dose, and compared with camrelizumab combined with apatinib 125mg, QD orally, apatinib 250mg, QD, it has more therapeutic advantages. Compared with camrelizumab combined with apatinib 375mg, QD orally, apatinib 250mg, QD, it has better tolerance. In a phase Ⅱ study of camrelizumab combined with apatinib for non-squamous and non-small cell lung cancer, grade 3 rash was observed in multiple subjects during the tolerability observation period at the 375mg dose level of camrelizumab in combination with apatinib and multiple subjects withdrew the study drug due to adverse events during the tolerability observation period. In a phase Ⅱ study of camrelizumab combined with apatinib for advanced primary liver cancer (enrolled patients with hepatocellular carcinoma and intrahepatic cholangiocarcinoma), grade 3 thrombocytopenia was observed in multiple subjects during the tolerability observation period at the 375mg dose level of camrelizumab in combination with apatinib.

In addition, for the choice of HAIC medication, the existing reports in the Journal of Hepatology and JAMA Oncology all use the mFOLFOX6 perfusion regimen, that is, oxaliplatin 85mg/m^2^, calcium folinate 400mg/m^2^, 5-fluorouracil 400mg/m^2^ intravenous bolus on the first day, and continuous infusion of 5-fluorouracil at a dose of 2400mg/m^2^ for the subsequent 46 hours. However, the EACH study that introduces the mFOLFOX6 regimen into the treatment of liver cancer used systemic intravenous chemotherapy. Considering that 5-fluorouracil is metabolized rapidly in the body, it is necessary to give a continuous maintenance dose after the intravenous bolus reaches an effective blood concentration. But HAIC is administered directly through the artery, the drug directly enters the tumor area, and no initial bolus dose is required to achieve an effective systemic steady-state concentration of 5-fluorouracil. Therefore, we further simplified the mFOLFOX6 regimen to a mFOLFOX7 regimen of 85mg/m^2^ oxaliplatin, 400mg/m^2^ leucovorin and 2500mg/m^2^ 5-fluorouracil maintained for 46 hours. In addition, the HAIC combined with standard-dose sorafenib regimen published by Professor Shi Ming’s team in JAMA Oncology has increased drug toxicity but can be tolerated.

Therefore, in this study, oxaliplatin 85mg/m^2^, leucovorin 400mg/m^2^, and 5-fluorouracil 2500mg/m^2^ for 46 hours continuous infusion, camrelizumab 200mg fixed dose and Q3W combined with apatinib 250mg QD taken orally will be selected. This study aimed to evaluate the efficacy and safety of combined therapy in patients with BCLC stage C advanced hepatocellular carcinoma.

## 3.3 Sample Size Estimation

This is a prospective exploratory study which intends to adopt the Simon two-stage study design. According to previous studies, the expected effective rate of the new treatment could achieve 60.8%, and the poor effective rate is set at 40%. To control type II error rate no more than 0.20 at a one-sided significance level of 0.025 and, the two-stage design requires 26 patients to be enrolled in the first stage, the study treatment will be considered non-effective and study will be terminated if no more than 11 patients (< 12 patients) achieve CR/PR, otherwise another 21 patients will be enrolled. The study treatment will be considered with promising effectiveness if more than 25 patients in total achieve CR/PR..

## 3.4 Ways to Reduce Bias

### 3.4.1 Enrollment/Randomization/Blinding Steps

This study is a single-arm trial, with sequential enrollment, no randomization, and no blinding.

### 3.4.2 Blind Evaluation

Not applicable.

### 3.4.3 Break the Blind

Not applicable.

# 4. SUBJECT SELECTION AND WITHDRAWAL

## 4.1 Inclusion Criteria

Patients must meet all of the following inclusion criteria to be enrolled in this study:

1. The patients voluntarily join the study and sign the informed consent;

2. Age ≥18 years and ≤70 years, both male and female;

3. Clinically or pathologically confirmed BCLC stage C hepatocellular carcinoma without prior TACE or systemic therapy;

4. At least one evaluable lesion in the liver, which is the predominant tumor burden;

5. Child-Pugh score ≤ 7 (Child-Pugh A-B);

6. Liver tumor burden does not exceed 50% of liver volume;

7. Can swallow pills normally;

8. ECOG score:0~1 (the ECOG scoring criteria are in the appendix);

9. With a life expectancy of ≥ 12 weeks;

10. The function of major organs meet the following requirements (without use of any blood components, cell growth factors and other corrective drugs within 14 days of the first dose):

- Absolute neutrophil count ≥ 3.0×10^9^/L;
- Platelet ≥ 80×10^9^/L;
- Hemoglobin ≥ 90 g/L;
- Serum albumin ≥ 28 g/L;
- Thyroid stimulating hormone (TSH) ≤ 1×ULN (If abnormal, FT3 and FT4 levels should be investigated at the same time. If FT3 and FT4 levels are normal, they can be included in the group);
- Bilirubin ≤ 1.5×ULN (7 days before first medication);
- ALT & AST ≤ 3×ULN (7 days before first medication);
- AKP ≤ 2.5×ULN; Serum creatinine ≤ 1.5×ULN;

11. Female patients who are non-surgical sterilized or at childbearing age need to use medically-approved contraceptive method (such as an IUD, birth control pill, or condom) during the study treatment and for 3 months after the end of the study treatment period. Non-surgical sterilized female patients of childbearing age must have a negative serum or urine HCG test within 72 hours before study enrollment; non-surgical sterilized female patients of childbearing age must be non-breastfeeding. For male patients whose mates are women of childbearing potential, effective contraception should be used during the trial and for 3 months after the last dose of camrelizumab.

## 4.2 Exclusion Criteria

1. Patients with any active autoimmune disease or history of autoimmune disease (such as, but not limited to: autoimmune hepatitis, interstitial pneumonia, uveitis, enteritis, hepatitis, hypophysitis, vasculitis, nephritis, hyperthyroidism; patients with vitiligo; complete remission of asthma in childhood without any intervention in adulthood can be included; patients with asthma requiring medical intervention with bronchodilators cannot be included);

2. The patients are using immunosuppressant or systemic hormone therapy to achieve the purpose of immunosuppression (dose > 10mg/day prednisone or other equivalent efficacy hormone), and continues to use it within 2 weeks before enrollment;

3. Severe allergic reactions to other monoclonal antibodies;

4. Known history of CNS metastases or hepatic encephalopathy;

5. Those with a history of organ transplantation;

6. Ascites with clinical symptoms, those who need puncture, drainage, or those who have received ascites drainage within the past 3 months, except those who only have a small amount of ascites on imaging but no clinical symptoms;

7. Hypertension that is not well controlled with anti-hypertensive medication (systolic blood pressure ≥140 mmHg or diastolic blood pressure ≥90 mmHg);

8. Those who have clinical symptoms or diseases of the heart that are not well controlled, such as (1) NYHA class 2 or higher heart failure; (2) unstable angina pectoris; (3) myocardial infarction within 1 year; (4) clinically significant supraventricular or ventricular arrhythmias requiring treatment or intervention; (5) Tc > 450ms (male); QTc > 470ms (female);

9. Patients with abnormal coagulation function (INR>2.0, PT>16s), bleeding tendency or receiving thrombolytic or anticoagulant therapy, allowing prophylactic use of low dose aspirin and low molecular heparin;

10. Those who have clinically significant bleeding symptoms or a clear bleeding tendency within 3 months before enrollment, such as whooping cough/hemoptysis 2.5ml or more, gastrointestinal bleeding, esophagus and gastric varices with bleeding risk, hemorrhagic gastric ulcer or suffering from vasculitis, etc. If the fecal occult blood is positive during the baseline period, it can be re-examined. If it is still positive after the re-examination, gastroscopy is required. If the gastroscopy shows severe esophagogastric varices, the patients cannot be included in the group (except those who have undergone gastroscopy within 3 months before enrollment to exclude such conditions);

11. Arterial/venous thrombotic events, such as cerebrovascular accident (including transient ischemic attack, cerebral hemorrhage, cerebral infarction), deep venous thrombosis and pulmonary embolism, occur within 6 months before enrollment;

12. Known hereditary or acquired predisposition to bleeding and thrombosis (such as hemophilia, coagulopathy, thrombocytopenia, etc.);

13. Urine routine indicates urine protein ≥++ and confirmed 24-hour urine protein amount > 1.0 g;

14. The patients with active infection, unexplained fever ≥ 38.5℃ within 7 days before medication, or baseline white blood cell count > 15×10^9^/L;

15. The patients with congenital or acquired immunodeficiency (such as HIV-infected patients);

16. HBV-DNA > 2000 IU/ml (or 10^4^ copies/ml); or HCV-RNA > 10^3^ copies/ml; or HBsAg + anti-HCV antibody positive patients;

17. The patients have other malignant tumors in the past 3 years or at the same time (except for cured basal cell carcinoma of the skin and carcinoma in situ of the cervix);

18. The patients with bone metastases who received palliative radiotherapy to > 5% of the bone marrow area in the 4 weeks prior to participation;

19. The patients has previously received other anti-PD-1 antibody therapy or other immunotherapy targeting PD-1/PD-L1, or previously received apatinib therapy;

20. Those who received live vaccines less than 4 weeks before study medication or

who may receive life vaccines during the study period.

21. Pregnant or breastfeeding women, or women of childbearing age who are reluctant to use contraception;

22. According to the judgment of the investigator, the patient has other factors that may affect the results of the study or cause the study to be terminated halfway, such as alcoholism, drug abuse, other serious diseases (including mental diseases) that require concomitant treatment, and serious laboratory abnormalities, with family or social and other factors, which will affect the safety of patients.

## 4.3 Subjects Withdraw from Study or Discontinue Study Treatment

### 4.3.1 Criteria for Discontinuing Treatment

If one or more of the following conditions occur, the subject must withdraw/discontinue treatment:

1. Subject withdraws informed consent and asks to withdraw;
2. Progression on imaging studies;

If the disease progresses for the first time according to RECIST (version 1.1), it needs to be confirmed after 4-6 weeks (except for those with rapid progression and significant clinical progression);

Subjects with confirmed disease progression, if the clinical symptoms are stable, they can continue to receive treatment according to the judgment of the investigator until another imaging progression occurs;

Clinically stable definition: a. No significant clinical symptoms, laboratory test indicators change; b. No change in performance status score (worsening); c. Rapid progression of non-tumor and tumor progression not involving vital organs/sites (such as spinal cord compression);

1. The cumulative use of camrelizumab reaches 2 years (no imaging progress), and subjects who are confirmed to be CR by imaging examinations can consider discontinuing the drug after 12 cycles of treatment;
2. Those who cannot tolerate toxicity;
3. Those with poor compliance;
4. Those lost follow-up or pregnancy;
5. Other circumstances that the investigator considers necessary to withdraw from the study.

### 4.3.2 Steps to Withdraw From the Study or Discontinue Study Treatment

It is necessary to make every effort to complete the efficacy and safety checks at the time of withdrawal as specified in the protocol, as well as to complete the safety follow-up period, and to comprehensively record adverse events (AEs) and outcomes. The researchers can suggest or provide new or alternative treatment methods to the subjects according to the actual conditions of the subjects. Patients without disease progression should continue to be followed up for imaging evaluation until the subjects start new anti-tumor therapy or disease progression.

If the subject refuses further visit to the research center, their survival status should continue to be tracked unless the subject withdraws consent to disclose information or to be contacted further. In these cases, no further study evaluations should be conducted and no further data should be collected.

## 4.4 Study Termination Criteria

The study termination criteria are not limited to be following:

1. Unexpected, significant or unacceptable risk to the patient is identified;
2. During the execution of the test, it is found that there are major mistakes in the plan;
3. The study drug/trial treatment is ineffective, or there is no point in continuing the trial;
4. The sponsor decides to discontinue study due to reasons such as severe lag in patient enrollment or frequent protocol deviations.

## 4.5 End-of-trial Definition

Statistical analysis of the primary and secondary endpoints of the study will be performed at the end of the study 6 months after the last patient’s first medication.

All patients will be followed up to 12 to 24 months after the last patient’s medication, and supplementary analysis of the primary and secondary endpoints of the study will be performed after the end of follow-up.

After the end of the study, if patients continue to benefit, they can continue to use the study drug for 1 year (unless the treatment discontinuation criteria are met), and the occurrence of SAEs will be collected and recorded during the treatment period and after the last medication according to the protocol.

# 5. STUDY DRUGS

## 5.1 Overview of Study Drugs

### 5.1.1 The Way to Get Drugs

The drug 1 will be provided free of charge by the co-organizer (Jiangsu Hengrui Pharmaceutical Co., Ltd.), and experimental drug 2 will be provided by the co-organizer (Jiangsu Hengrui Pharmaceutical Co., Ltd.) at the discount on the principle of buying 3 boxes and getting 3 boxes free (250mg*10# in one box). The study drugs will be uniformly packaged and passes the inspection (refer to the corresponding inspection report).

Concomitant medications for adverse events, as well as prophylactic medications, are not study drugs and will not be provided by the sponsor. These medicines are marketed products procured by research centers and will be kept according to the package insert or summary of product characteristics.

### 5.1.2 Dosage Form, Appearance, Packaging and Label of Drugs

Test drug 1: Camrelizumab for Injection

Manufacturer: Suzhou Shengdiya Bio-pharmaceutical Co., Ltd. (a subsidiary of Jiangsu Hengrui Pharmaceutical Co., Ltd.)

Dosage form: freeze-dried powder

Specifications: The specification of this product is tentatively set at 200mg, packaged in a 20ml vial.

Lot number: refer to drug label

Usage: intravenous drip

Validity period: From the production date, the shelf life is tentatively set for 2 years.

Storage conditions: sealed and protected from light, it should be placed in a medical refrigerator at 2 to 8 ℃ during storage, and this product should not be frozen.

Test Drug 2: Apatinib Mesylate Tablets

Manufacturer: Jiangsu Hengrui Pharmaceutical Co., Ltd.

Dosage form: tablet

Specification: 250mg/tablet

Lot number: refer to drug label

Usage: oral

Validity period: From the production date, the expiration date is tentatively set for 2 years.

Storage conditions: shading, sealed, and it should be stored below 25 ℃.

Specification and quantity of camrelizumab drug packaging: 200mg/20ml vial, 1 per small box, 20 small boxes in each large box.

The labeling of the study drug will be carried out in accordance with the relevant regulations and guidelines of GCP (Good Clinical Practice for Drugs), and the contents of the label include but are not limited to: clinical approval number, research name, drug name, drug number, packaging specification, production batch number, expiration date, usage and dosage, drug storage conditions, and the words “for clinical research only” indicated.

### 5.1.3 Preservation and Stability of Drugs

The researcher, or his authorized representative (such as a pharmacist), will ensure that all study drugs are stored in a secure, controlled-access area that complies with storage conditions, and that they are stored in compliance with applicable regulatory requirements.

The study drugs should be stored in its original container and in accordance with the drug label. In case of inconsistency between the storage conditions on the label and other information of the test drug (such as the drug manual), the storage conditions on the label shall prevail.

Research center must be able to measure and record daily maximum and minimum temperatures in all storage locations (such as frozen, refrigerated, or room temperature). The recording period should begin with receipt of the study drugs and continue until the end of the study. Even with a continuous monitoring system, the research center should have logs to ensure correct storage temperatures. Temperature monitoring devices and storage devices (such as refrigerators) should be checked regularly to ensure proper functioning.

In the event of any deviation from the product labeling conditions, it should be reported in a timely manner. The research center should actively take measures to return the study drugs to the storage conditions stated on the label as soon as possible, and at the same time, report the temperature deviation and the measures taken to the sponsor.

The experimental drugs affected by temperature deviation need to be temporarily isolated until it is allowed to continue to be used by the sponsor, and it is not a protocol deviation. Continued use of the affected study drugs without the sponsor’s permission is a protocol deviation. Sponsors will provide the research center with specific steps for reporting temperature deviations.

### 5.1.4 Preparations of Drugs

Camrelizumab should be prepared by a qualified or experienced researcher according to the drug manual, the package insert of the marketed drug, such as a doctor, pharmacist or medical assistant (as permitted by the national or research unit operation instructions).

Please refer to the camrelizumab drug handbook for the mixing method and concentration (preparation) of the dosing solution and its usage. Since this product does not contain any anti-microbial preservatives and bacteriostatic agent, care must be taken to ensure that the prepared solution is sterile.

The total storage time (the total time stored in the refrigerator and at room temperature) from the preparation of camrelizumab to administration should not be exceed 24 hours. Refer to the medication handbook for details on the storage and use time of the prepared drug at room temperature/light and in the refrigerator.

The expired or leftovers must be discarded.

### 5.1.5 Usage of Drugs

Camrelizumab is an intravenous injection and must be administered by qualified or experienced researchers in the outpatient clinic or ward of the research center, and must not be taken out of the research center for use.

Within 72 hours before each medication, except imaging examinations, subjects must complete various clinical examinations to assess the tolerance of continued medication.

Intravenous camrelizumab for 30 minutes (not less than 20 minutes and not more than 60 minutes, including washout phase). Intravenous and bolus injections are not allowed.

Intravenous infusion is accomplished through a medical infusion bag using an infusion set with an in-line filter (0.2μm).

Do not use this infusion line to give other drugs before or after the infusion.

Apatinib mesylate tablets are oral preparations. The researchers will prescribe the patients, and the patients will take the medicine home and take it orally after meals. For details, refer to the dosing scheme.

### 5.1.6 Special Dosing Equipment Considerations

The research center will also obtain IV bags, diluents, and micron-sized filters (such as 0.2/1.2μm. Refer to the drug handbook for required filters).

## 5.2 Treatment Plan

Eligible patients were administrated with up to 6 cycles of HAIC-FOLFOX. Meanwhile, all patients were treated with camrelizumab and apatinib until disease progression or unacceptable toxicities.

Chemotherapy with mFOLFOX regimen through hepatic artery infusion, namely HAIC, will perform celiac trunk and superior mesenteric artery angiography after successful percutaneous hepatic artery cannulation under DSA guidance. After the arterial catheter is cannulated to the predetermined position, the patient with indwelling catheter will be sent to the ward. In the ward, the catheter will be conducted to a syringe pump and the following chemotherapy drugs are continuously pumped: oxaliplatin 85mg/m^2^ for 2 hours, leucovorin 400mg/m^2^ for 2 hours, 5-fluorouracil 2500mg/m^2^ for 46 hours. After chemotherapy, the catheter will be removed and the bleeding will be stopped by pressure bandage for 12 hours.

Camrelizumab will be administered intravenously at a fixed dose of 200mg, or 3mg/kg for those with body weight<50 kg at baseline, administered by intravenous drip (no preventive medication is required), each infusion will be 30 minutes (not less than 20 min, not more than 60 min), starting on day 4 in the second HAIC cycle, administered once every 3 weeks, and the longest cumulative medication period will be 2 years.

During the study period, camrelizumab will be administered once every 3 weeks, and the dosing window period is ±3 days from the planned dosing time. If the delayed administration of camrelizumab exceeds the planned administration time for 3 days, the administration will not be administered at that time, and the original administration dose will be continued until the next planned administration time.

Apatinib will be taken orally after meals, starting on day 8 in the first HAIC cycle, once daily, 1 tablet (250mg/tablet) each time, 3 weeks as a cycle.

Definition of postprandial administration: administration within 30 minutes after the end of the meal.

Patients will be treated with the study drug until protocol-specified treatment discontinuation criteria occur.

## 5.3 Dose Adjustment

During the study period, the dose of the study drugs can be adjusted according to the toxic and side effects of the study drugs, including: dose suspension, down-regulation, adjustment of the mode of administration and dose termination.

Combination therapy was discontinued when disease progression, disease downstaging to have an opportunity to perform surgery, unacceptable toxicities, or death occurred. In the event of grade ≥3 or serious treatment-related adverse events (TRAEs), the related study treatment should be discontinued, and the other two were allowed to continue as determined by the investigator. When the FOLFOX infusion caused TRAEs, the HAIC was allowed to delay.

Dose suspensions for camrelizumab will be permitted during the study, with drug suspensions of up to 12 weeks allowed.

In addition to dose suspension, for subjects whose body weight loses to under 50 kg during the treatment period, camrelizumab can be reduced to 3mg/kg, Q3W administration as appropriate.

Dose adjustments due to apatinib-related toxicity will include: dose suspension, dose mode adjustment (first adjustment: 5 days on and 2 days off; second adjustment: 7 days on and 7 days off), and dose termination. Adjustment in apatinib dose during the study period will no longer allow callbacks.

If AEs clearly related to apatinib occur during the trial, such as hypertension, proteinuria, hand-foot syndrome, etc., apatinib can be suspended. After the toxicity recovers, administer the original dose, adjust the mode of administration, or terminate the dose as appropriate. After the termination of apatinib administration, the subjects can continue to use camrelizumab monotherapy.

If immune-related toxicity occurs during the trial, such as immune pneumonia, hepatitis, colitis, etc., camrelizumab and apatinib should be suspended as appropriate, and the drugs can be resumed when the toxicity returns to ≤ grade 1 or baseline level (for those with abnormal baseline laboratory indications such as ALT/AST and TBIL). When resuming medication, camrelizumab should be resumed first, and the medication should be observed.

After 7-14 days of no significant abnormality, the administration of apatinib will be started again, and the dose of the subsequent administration of apatinib will be adjusted.

During the trial, if ≥grade 3 immune pneumonia, ≥grade 3 TBIL increase (recurrence), grade 4 ALT/AST increase (recurrence), and other grade 4 immune-related toxicity reactions (except hypothyroidism), grade 4 injection reaction occur, or if camrelizumab cannot be recovered to ≤ grade 1 or baseline level after suspending camrelizumab for more than 12 weeks due to immune-related toxicity (abnormal baseline period), camrelizumab must be permanently discontinued.

After the administration of camrelizumab is terminated, if the investigator judges that the subject can benefit from apatinib monotherapy, the subject will be allowed to receive apatinib monotherapy after the toxicity recovers, until an event that meets the protocol-specified treatment discontinuation criteria occurs.

If capillary hyperplasia of grade 3 or above occurs during the trial, no dose adjustment will be required for apatinib, and camrelizumab will be suspended until the toxicity returns to grade 2 or below.

Symptoms/signs or abnormal laboratory indicators in the subjects during the trial should be treated in time, and it is recommended to refer to the following table for corresponding dose adjustment:

**Medication Dosage Adjustment Form**

| Drug-related Toxicity | | Grade | Dosage Pause | | Restoring dosing standards | Apatinib dose adjustment method | Criteria for discontinuation of medication |
| --- | --- | --- | --- | --- | --- | --- | --- |
|  |  |  | Camrelizumab | Apatinib |  |  |  |
| Toxicity associated with camrelizumab and apatinib | Hematological toxicity | Grade 1 & 2 | No | No | - | - | - |
|  |  | Grade 3 | No | Yes (except for decreased lymphocyte count) | Wait until the toxicity returns to ≤ grade 2 | Use the original dose, if grade 3 hematological toxicity occurs again, adjust the apatinib dosing method. | - |
|  |  | Grade 4 | Yes | Yes | Wait until the toxicity returns to ≤ grade 2 | The first time: adjust to 5 days of medication and 2 days of withdrawal;  again: adjust to 7 days of medication and 7 days of withdrawal | If grade 3 or higher hematologic toxicity recurs after two adjustments, discontinue apatinib administration. |
|  | Immune-associated pneumonia | Grade 2 | Yes | No | Wait until the toxicity returns to ≤ grade 1 | The first time: the original dose is resumed;  the second time: adjust to 5 days of medication and 2 days of withdrawal;  the third time: adjust to 7 days of medication and 7 days of withdrawal | Discontinue camrelizumab administration if it cannot recover to ≤ grade 1 after discontinuation for > 12 weeks. |
|  | Elevated ALT or AST | Normal at baseline: grade 2;  Grade 1 at baseline: grade 3 | Yes (Unable to recover to ≤ grade 1 after receiving hepatoprotective therapy) | Yes | Wait until the toxicity returns to ≤ grade 1 | The first time: adjust to 5 days of medication and 2 days of withdrawal;  the second time: adjust to 7 days of medication and 7 days of withdrawal | 1.Discontinue camrelizumab administration if it cannot recover to ≤ grade 1 after discontinuation for > 12 weeks;  2.If grade 4 KT/AST elevation occurs again, discontinue camrelizumab administration. |
|  |  | Normal at baseline: ≥ grade 3;  Grade 1 at baseline: grade 4 | Yes | Yes | Wait until the toxicity returns to ≤ grade 1 | The first time: the original dose is resumed;  the second time: the drug is adjusted to 5 days of medication and 2 days of withdrawal;  the third time: the drug is adjusted to 7 days of medication and 7 days of withdrawal |  |
|  | Elevated TBIL | Grade 2 | Yes | Yes | Wait until the toxicity returns to ≤ grade 1 | The first time: the original dose is resumed;  the second time: adjust to 5 days of medication and 2 days of withdrawal;  the third time: adjust to 7 days of medication and 7 days of withdrawal | 1.Discontinue camrelizumab administration if it cannot recover to ≤ grade 1 after discontinuation for > 12 weeks;  2.If ≥ grade 3 TBIL elevation occurs again, discontinue camrelizumab administration. |
|  |  | ≥ grade 3 | Yes | Yes | Wait until the toxicity returns to ≤ grade 1 | The first time: the original dose is resumed;  the second time: adjust to 5 days of medication and 2 days of withdrawal;  the third time: adjust to 7 days of medication and 7 days of withdrawal |  |
|  | Other non-hematological toxicities | Grade 1 | No | No | - | - | - |
|  |  | Grade 2（lasting≥7d） | Yes | Yes | Wait until the toxicity returns to ≤ grade 1 | Original dose | Discontinue camrelizumab administration if it cannot recover to ≤ grade 1 after discontinuation for > 12 weeks. |
|  |  | Grade 3 | Yes | Yes | Wait until the toxicity returns to ≤ grade 1 | The first time: adjust to 5 days of medication and 2 days of withdrawal;  the second time: adjust to 7 days of medication and 7 days of withdrawal |  |
| Toxicity associated with camrelizumab | Capillary hemangioma | Grade 3 | Yes | No | Wait until the toxicity returns to ≤ grade 2 | Original dose | Discontinue camrelizumab administration if it cannot recover to ≤ grade 1 after discontinuation for > 12 weeks. |
| Toxicity associated with apatinib | Hypertension | Grade 3 (after corrective treatment) | No | Yes | Wait until the toxicity returns to ≤ grade 1 | The first time: the original dose is resumed;  grade 3 hypertension occurs again: adjust to 5 days of medication and 2 days of withdrawal;  grade 3 hypertension occurs the third time: adjust to 7 days of medication and 7 days of withdrawal | If grade 3 hypertension recurs after two adjustments, discontinue apatinib administration. |
|  |  | Hypertensive crisis | Yes | Yes | Wait until the toxicity returns to ≤ grade 1 | Permanently discontinue apatinib administration. | Discontinue apatinib administration. |
|  | Proteinuria (without a significant increase in serum creatinine) | Grade 3 (24h urine protein quantification) | No | Yes | Wait until the toxicity returns to ≤ grade 2 | Apatinib is adjusted to 5 days of medication and 2 days of withdrawal and if grade 3 proteinuria occurs again, apatinib is adjusted to 7 days of medication and 7 days of withdrawal. | Discontinue apatinib administration if grade 3 proteinuria occurs after two adjustments. |
|  | Hand-foot syndrome | Grade 3 | No | Yes | Wait until the toxicity returns to ≤ grade 1 | Apatinib is adjusted to 5 days of medication and 2 days of withdrawal and if grade 3 hand-foot syndrome occurs again, apatinib is adjusted to 7 days of medication and 7 days of withdrawal. | Discontinue apatinib administration if grade 3 hand-foot syndrome occurs after two adjustments. |
|  | Headache | Grade 2 headache lasting ≥7 days after symptomatic treatment, or grade 3 headache | No | Yes | Wait until the toxicity returns to ≤ grade 1 | Apatinib is adjusted to 5 days of medication and 2 days of withdrawal and if it occurs again, apatinib is adjusted to 7 days of medication and 7 days of withdrawal. | Discontinue apatinib administration if it occurs after two adjustments. |
| The subjects experience significant toxicity that persists after symptomatic treatment during the trial, including grade 2 non-hematological toxicity (except asymptomatic grade 2 hypertension) that persist for 2 weeks or more, abnormal laboratory test indicators (except <2g/24h proteinuria), investigators may consider adjusting the dosing method of apatinib in the follow-up study phase after suspending the drug and waiting for the toxicity to recover according to the tolerance of the subjects.  During the study, the investigators can give appropriate dose adjustments in the event of comprehensive drug-related toxicity of the subjects in combination with the above-mentioned dose adjustment regulation (for example, if the subject has multiple grade 2 study drug-related toxicity and tolerates the study drug poorly, the administration of apatinib can be adjusted after the drug is suspended and the toxicity recovers).  During the trial, if the subject develops fever (>38℃) and requires corrective medication, or has obvious symptoms of wheezing, shortness of breath, and suffocation, the current or next planned camrelizumab administration should be discontinue until the symptoms recover. After the symptoms are relieved and stabilized for more than 7 days, the drug will be administered according to the follow-up schedule of camrelizumab administration, and if necessary, pneumonia should be excluded by imaging examination before administration.  During the trial, once the occurrence of: hypertensive crisis, cerebral hemorrhage, ≥grade Ⅱ pulmonary hemorrhage, ≥grade Ⅲ other hemorrhage, arterial thrombosis, grade Ⅳ venous thrombosis, leukoencephalopathy syndrome, gastrointestinal perforation, the subject should discontinue apatinib administration, suspend the administration of camrelizumab, and take active symptomatic treatment, and whether to continue camrelizumab treatment will depend on the recovery of the subject’s toxicity. | | | | | | | |

## 5.4 Management, Distribution and Recovery of Drugs

Special personnel of each research center will be responsible for the management, distribution and recovery of the study drugs in this study. The investigators must ensure that all experimental drugs are only used for the subjects participating in this study, and their dosage and usage should follow the research protocol. The remaining study drug, camrelizumab, will be returned to the co-sponsors. Expired or remaining liquid medicines will be destroyed directly according to medical waste standards. The study drugs should not be transferred to any non-clinical study participant.

The test drugs should be stored according to the label requirements. When the drugs arrive at the research center, the drug receipt form must be signed in duplicate, and the clinical research unit and the co-organizer each hold one copy. At the end of the study, the remaining drugs and empty boxes need to be recovered, and both parties should sign in a drug recovery form. The distribution and recovery of each drug should be recorded in a timely manner on a special record sheet.

Inspectors will be responsible for supervising the supply, use, storage and disposal of remaining drugs for clinical trials. The used experimental drugs can be recycled and destroyed by the company, or the research center can be entrusted to destroy. Before entrusting a research center to destroy a drug, the inspector needs to confirm the research center’s destruction process and have relevant instructions, and ensure that there is a relevant destruction certificate after destruction.

## 5.5 Concomitant Therapy

Concomitant treatments are other treatments given at the discretion of the investigators based on the interests of the subjects.

The concomitant medication and concomitant treatment of subjects 30 days before the start of medication and during the study period should be recorded in the eCRF in strict accordance with the requirements of GCP.

Once a subject discontinues trial treatment, only concomitant medications and concomitant treatments used for new or unresolved adverse events related to trial treatment should be recorded for at least 30 days after the last dose.

### 5.5.1 Other Anti-tumor/Anti-cancer or Study Drugs

No other anti-tumor therapy will be permitted while the subject is receiving study drugs.

It is not allowed to take the listed anti-tumor Chinese medicines at the same time: Carbocisteine, YaDanZiYouRuanJiaoNang, Zhemu syrup, Cantharides, Cinobufotalin, Toad venom, Kang’ai injection, Kanglaite, Zhongjiefeng injection, Aidi injection, Aweihuapi Plaster, Kang’aiping tablet, Fukang capsule, Xiaoaiping, Pingxiao capsule, Pingxiao pill, Shendan Sanjie capsule, Ankangxin capsule, Bosheng’aining, Zedoary turmeric oil glucose injection, Kanglixin capsule, Cidan capsule, Ganfule tablet, Huaier granule, Delisheng injection, and other traditional Chinese medicine preparations with “anti-tumor” effect clearly stated in the instructions.

Palliative radiotherapy will be permitted for the treatment of painful bone lesions in the study, provided these lesions are known to be present at the time of enrollment, and the investigator clearly indicates that the use of palliative radiotherapy does not imply disease progression. Patients can receive bisphosphonates for the treatment of bone metastases. If systemic therapy or topical analgesia cannot effectively control the pain of bone metastases, palliative radiotherapy will be allowed to be used in a small area (the radiotherapy area must be less than 5% of the bone marrow area, and the percentage of bone marrow content in the human body is shown in the figure in appendix 3).

During the trial, palliative treatment for lesions other than the liver and lungs will be allowed (when the patient needs to receive treatment to improve symptoms due to disease progression), and the treatment will include the treatment of hydrothorax and ascites, etc. During the treatment period, the patient should suspend the trial drugs until the end of the palliative care recovery period.

### 5.5.2 Supportive Care

Palliative and supportive care for disease-related symptoms will depend on the judgment of the investigator and relevant guidelines. Hepatitis B treatment with optimal oral antiviral drugs will be allowed during the trial, but antiviral treatment with interferon is not allowed.

During treatment, subjects can be given the best supportive care. The clinical comorbidities and various adverse reactions should be actively treated and dealt with, especially the adverse reactions related to immunity.

### 5.5.3 Immune Preparation

Concomitant use of thymosin, interferon, interleukin-2 and other immune agents will not be allowed.

### 5.5.4 Drugs That May Interact with Apatinib

In vitro studies have shown that apatinib is mainly metabolized by the hepatic P450 enzyme CYP3A4, and has a strong inhibitory effect on CYP3A4 and CYP2C9, and a moderate inhibitory effect on CYP2C19. CYP3A4 inducers (dexamethasone, catamirazine, rifampicin and phenobarbital) should be used with caution and strong CYP3A4 inhibitors (ketoconazole, itraconazole, erythromycin, and clarithromycin) will be contraindicated.

CYP3A4 substrates that will be contraindicated during the trial (drugs with a narrow safety window and the possibility of serious adverse reactions after metabolism is affected) include but are not limited to:

- Hypoglycemic drugs: tolbutamide, chlorpropamide;
- Ergot derivatives: dihydroergotamine, ergometrine, ergotamine, methylergometrine (potential risk of ergot toxicity, including severe vasospasm leading to peripheral and cerebral ischemia);
- Antipsychotics: pimozide (potentially increases the risk of QT prolongation);
- Antiarrhythmics: amiodarone (6 months before randomization), bepridil, flecainide, lidocaine, mexiletine, quinidine, propafenone;
- Immunomodulators: cyclosporine, tacrolimus, sirolimus (potentially increases the risk of nephrotoxicity and neurotoxicity);
- Others: quetiapine, risperidone, clozapine, atomoxetine hydrochloride;

If warfarin is used for anticoagulation during the trial, dose reduction should be considered, and close monitoring should be considered. Discontinuation of the trial drug should be considered if necessary.

### 5.5.5 Drugs That Cause Cardiac QT Prolongation

Due to the toxic and side effects of clinically prolonging the QT interval of tinib drugs, the drugs that prolong the QT interval should be used with caution during the experimental study. It mainly includes but is not limited to several types of drugs listed below:

- Antibiotics: Fluroquinolones: sparfloxacin, gatifloxacin, levofloxacin, moxifloxacin, ofloxacin, ciprofloxacin; macrolides: erythromycin, clarithromycin, telithromycin, azithromycin, roxithromycin, metronidazole;
- Antiarrhythmics: quinidine, procainamide, disopyramide, flecainide, propafenone, amiodarone, dronedarone, sotalol, dofetilide, ibutilide;
- Angina pain relievers: ranolazine, ivabradine;
- Antipsychotics: risperidone, fluphenazine, droperidol, haloperidol, thioridazine, pimozide, olanzapine, clozapine;
- Antifungal drugs: voriconazole, posaconazole;
- Antimalarial drugs: mefloquine, chloroquine;
- Antihistamines: terfenadine, astemizole, hydroxyzine;
- Gastrointestinal drugs: antiemetics: ondansetron, granisetron, dorasetron, droperidol (0.625 to 1.25 mg may be safe), hydroxyzine; prokinetics: cisapride, domperidone, metoclopramide;
- Antidepressants: amitriptyline, imipramine, clomipramine, dothiepin, doxepin.

### 5.5.6 Surgery or Palliative Radiotherapy

Any surgery or palliative radiotherapy during the study period should have its rationale and necessity. The interval between the treatment and the study drug should not affect the recovery of the wound and the search for the cause of unidentified bleeding as much as possible. It is recommended that study drug be suspended for 7 days before surgery or palliative radiotherapy, during the pre-treatment period, and for at least 7 days after the end of surgery/radiotherapy. Whether or not to retest dosing in subjects undergoing surgery will depend on clinical assessment of wound healing and postoperative recovery.

# 6. STUDY PROCEDURE

Patients must read and sign a current Ethics Committee (EC)-approved informed consent form before starting the study. The inspection and testing procedures will be carried out according to the time of the study flow table, and will not be affected by the length of the withdrawal time. However, it is allowed to change within the window period of various inspection items due to festivals, holidays or other management reasons.

## 6.1 Screening Assessment (Days D_-28_ to D_0_)

Signed informed consent from the patients should be obtained prior to all screening assessment steps. The following screening assessment procedures should be completed within the screening period (Days **_-28_** to **_0_**). Details of the schedule and types of these assessments are as follows:

【Sign Informed Consent】Before performing any clinical trial procedures, prior written consent must be obtained.

【Demographic data】gender, date of birth, ethnicity, height, weight;

【Tumor history】The main clinical symptoms and duration, basic imaging diagnosis (plain scan + enhanced CT or MRI) and laboratory test results before enrollment history of hepatitis and antiviral therapy and other related conditions of patients.

【Prior medical history】The name of the disease, the date of diagnosis, the name of the treatment drug, whether it is persistent, and the history of tumors other than hepatocellular carcinoma

【Concomitant medications】Record the concomitant medications and concomitant treatment within 30 days before starting medication and during the study period. Once the patient discontinues the trial treatment, only the concomitant medications and concomitant treatments used for new or unresolved adverse events related to the trial treatment should be recorded, until at least 30 days after the last medication. If the patient starts a new anti-tumor therapy within 30 days, only concomitant medications associated with the study drug and related adverse events are recorded.

【Thyroid function】FT3, FT4 and TSH;

【Pituitary adrenal axis examination】Including ACTH, cortisol, follicle stimulating hormone;

【HIV examination】HIV antibody examination;

【Hepatitis B and Hepatitis C examination】Hepatitis B two-and-a-half examination: If the examination results are abnormal, quantitative HBV DNA and HBsAg will be tested. Hepatitis C virus antibody (anti-HCV): If the patient tests positively for anti-HCV, HCV RNA detection should be carried out. Quantitative HBV DNA and HBV DNA will be tested every 2 cycles D_1_±7d and at the end of the study in patients with abnormal HBsAg at baseline.

【Alpha-fetoprotein】Determination of AFP;

【Adverse events】Adverse events will be recorded from the signing of informed consent, until at least 30 days after the last medication, and follow up until adverse events are relieved or stabilized. SAEs and irAEs should be followed up within 90 days after the last dose of camrelizumab. If the patients start new anti-tumor therapy, follow up until the start of tumor therapy (If the therapy starts within 30 days of the last medication, follow up until at least 30 days after the last medication. While if it starts more than 30 days after the last medication, follow up until the start of tumor therapy.).

【Imaging examinations】Including enhanced CT or MRI of the chest, abdomen and pelvis. During the screening period, subjects must also undergo enhanced MRI or enhanced CT of the brain. The baseline tumor evaluation in the screening period can be relaxed to within 3 weeks before treatment, and the CT/MRI results obtained before signing the informed consent can also be used for the tumor evaluation in the screening period as long as the requirements are met. Bone scans should be performed when clinically suspected bone metastasis.

【Biomarker specimen collection】For the enrolled cases confirmed by needle biopsy, the existing paraffin-embedded tumor tissue samples will be collected, or fresh biopsy specimens (soybean size) will be collected, with no less than 10 slices, of which will be collected 3 to 5 tumor sections with thickness of 3 to 5μm used for PD-L1 detection, and 5 to 8 paraffin sections with thickness of 8 to 10μm will be collected (paraffin rolls can be collected directly without climbing) or fresh biopsy specimens (soybean size) will be collected for the detection of biomarkers such as tumor mutational burden (TMB). Tumor sample collection and disposal methods are described in the laboratory manual. Unless otherwise specified, the following screening steps must be completed within 7 days of the first dose:

【Hematology】White blood cell count (WBC), neutrophil count (ANC), lymphocyte count (LYM), red blood cell count (RBC), hemoglobin (Hb), platelet count (PLT);

【Urinalysis】Urine protein, urine sugar, urine occult blood, urine erythrocytes and urine leukocytes. If the semi-quantitative method shows protein 2+ twice in a row, a 24-hour urine protein quantitative examination will be performed.

【Stool routine】If the fecal occult blood is positive, a re-examination will be required. After the re-examination, if the fecal occult blood is still positive, gastroscopy will be required.

【Blood biochemistry】Total bilirubin, direct bilirubin, ALT, AST, AKP, γ-GT, total protein, albumin, urea/urea nitrogen, creatinine, uric acid, fasting blood glucose, triglyceride, cholesterol, potassium, sodium, chloride, calcium, phosphorus, blood lipase (only during the screening period and when there are suspected symptoms of pancreatitis such as abdominal pain and abdominal distension), blood amylase (only during the screening period and when there are suspected symptoms of pancreatitis such as abdominal pain and abdominal distension);

【Coagulation function】Including INR, APTT, PT, FIB;

【Myocardial zymogram detection】

【Pregnancy test】Women of childbearing should perform urine pregnancy test 72 hours before the first drug use. If the urine pregnancy test is positive, the serum pregnancy test will be carried out, and the retest can be carried out to confirm if necessary.

【Vital signs】Temperature, pulse, respiratory rate, blood pressure;

【Blood pressure monitoring】During the screening period, the researcher or research nurse will measure the blood pressure of the patients. Every time the blood pressure is measured, smoking and drinking coffee are prohibited within 30 minutes before the measurement, and there is a quiet rest for at least 10 minutes. The measurement is taken in a sitting position. The elbow is placed at the same level as the heart and the blood pressure is measured on the same side for each measurement.

【Physical examination】General condition, head and face, skin, lymph nodes, eyes (sclera, pupil), ears, nose, throat, oral cavity, respiratory system, cardiovascular system, abdomen (including liver and spleen), reproductive-urinary system, musculoskeletal, nervous system, mental condition. Note: A comprehensive physical examination is required during the test, and only abnormal conditions are recorded in the eCRF. If there is no change compared with the screening period, there is no need to repeat recording.

【ECOG Score】Refer to Appendix 1.

【12-lead ECG】If there is any abnormality, it must be confirmed twice, or other necessary examinations should be added according to the judgment of the researcher.

【Echocardiography】

【Biomarker blood collection】10ml blood samples will be collected from patients during the baseline period, and will be processed and sent according to the laboratory manual.

The inclusion and exclusion criteria will be checked again, and only if the patients meet all the inclusion criteria and don’t meet the exclusion criteria can they be included in the study.

## 6.2 Treatment Visit

1.The first cycle

D_1_: Vital signs, physical examination and weight measurement, blood routine, blood biochemistry, urine routine, thyroid function test, pituitary-adrenal axis examination, start of hepatic arterial infusion chemotherapy, discharge after treatment, three weeks as a cycle, adverse events, concomitant medication;

D_7_: Blood routine, blood biochemistry, start oral apatinib, take it continuously, 3 weeks as a cycle, adverse events, concomitant medication;

D_21_: Vital signs, physical examination and weight measurement, blood routine, blood biochemistry, urine routine, thyroid function test, pituitary-adrenal axis examination, start of intravenous infusion of camrelizumab 200mg, adverse events, concomitant medication;

Within 24 hours after the first administration of camrelizumab, acute allergic reactions should be closely observed, and if they occur, the patients should be treated according to the hospital’s medical practice and relevant guidelines.

2.Subsequent cycles:

D_1_: Vital signs, physical examination and weight measurement, hepatic arterial infusion chemotherapy, discharge after treatment, three weeks as a cycle, adverse events, concomitant medication;

D_21_: Vital signs, physical examination and weight measurement, blood routine, blood biochemistry, urine routine, thyroid function test, pituitary-adrenal axis examination, start of intravenous infusion of camrelizumab 200mg, adverse events, concomitant medication;

Set a window of ±3 days, and the intravenous administration of camrelizumab should be performed after the examinations and tests specified in the flowchart have been completed and assessed.

【Imaging assessment】The imaging examination during the treatment period should be under the same conditions as the baseline examination (scanned slice thickness, use of contrast agent, etc.). The lesions found at the baseline should be examined every 2 cycles (Bone scan will be performed when bone progression is suspected or when CR is confirmed). If new lesions are suspected, they can be checked in time. The first PR/CR must be confirmed after 4 weeks ± 7 days. If the disease progresses for the first time according to RECISTv1.1, it needs to be confirmed by imaging examination after 4 to 6 weeks (except those with rapid progression and significant clinical progression).

The window period allowed by the imaging schedule will be ±7 days. Unplanned imaging studies may be performed when disease progression (such as worsening symptoms) is suspected.

【Thyroid function test】D_1_±7d in the second cycle, and then once every three cycles D_1_±7d;

【Alpha-fetoprotein】D_1_±7d every 2 cycles;

【Quantitative detection of HBV DNA and HBsAg】For patients with abnormal HBsAg at baseline, it will be performed every 2 cycles D_1_±7d during the test period.

【Blood pressure monitoring】During treatment, blood pressure monitoring will be completed by the patient and recorded in the patient diary card. In the first 2 cycles, blood pressure should be checked at least 3 times a week. If blood pressure is abnormal, follow up every day. If blood pressure is normal, blood pressure will be checked at least 2 times a week after 2 cycles. In addition, blood pressure will be remeasured by the researcher or research nurse at each follow-up visit.

## 6.3 End of Treatment/Withdrawal

If the patient has an event that meets the "4.3.1 Termination Criteria for Treatment", the treatment will end. At the end of study treatment or at the time of withdrawal from the study (set a +3d window period), if the patient has not been examined within 14 days before the end of the study, the patient should perform:

【Blood routine】【Blood biochemistry】【Urinalysis】【Stool routine】【Coagulation function】【Pregnancy test】【thyroid function test】【Alpha-fetoprotein】【Quantitative detection of HBV DNA and HBsAg】【Myocardial zymogram detection】【Vital signs】【Physical examination】【ECOG score】【ECG】【Echocardiography】【Blood pressure monitoring】【Adverse events】【Concomitant medication】【Apatinib drug recovery】

If no imaging examination is performed within 4 weeks before the end of treatment, it is necessary to perform an imaging examination at the end of the study treatment or when the study is withdrawn (set a +3d window period) to evaluate the efficacy. For patients with non-imaging evidence of progression (intolerable, other conditions), tumor assessments will be performed every 3 months until disease progression, death, or initiation of other anti-tumor therapy.

## 6.4 Follow-up

30 days after treatment termination/end (30 days ± 3d after the last study medication)

【Vital signs】【Physical examination】【ECOG score】【Blood routine】【Urinalysis】【Blood biochemistry】【Adverse events】【Concomitant medication】

Adverse events will be followed up until at least 30 days after the last study administration, and SAEs and irAEs will be followed up within 90 days after the last administration of camrelizumab. If the patients start new anti-tumor therapy, follow up until the start of tumor therapy (If the therapy starts within 30 days of the last medication, follow up until at least 30 days after the last medication. While if it starts more than 30 days after the last medication, follow up until the start of tumor therapy.).

【Survival follow-up】After the trial treatment is terminated, the survival status and subsequent anti-tumor treatment can be collected through clinical follow-up or telephone follow-up every 1 month until death.

# 7. STUDY ASSESSMENT

## 7.1 Evaluation of Effectiveness

### 7.1.1 Effectiveness Indicator

The primary efficacy endpoints of this study will be objective response rate (ORR) assessed by investigators based on RECIST 1.1 criteria.

Objective response rate (ORR): The proportion of subjects whose best overall response (BOR) reaches a complete response (CR) or a partial response (PR) according to the RECIST 1.1 criteria.

If the tumor assessment reaches CR or PR, it must be confirmed no less than 4 weeks (28 days) after the first occurrence of CR/PR.

For subjects with no documented progression or initiation of subsequent anti-tumor therapy, the best overall response will be determined based on all response assessments.

For subjects who continue camrelizumab after progression, the best overall response should be determined based on the response assessment recorded up to the time of initial RECIST 1.1-defined progression.

Secondary efficacy measures will include:

• Objective response rate as assessed by the investigator based on mRECIST criteria;

• Disease control rate (DCR): the percentage of participants who achieved a best overall response of CR, PR, or stable disease (SD) lasting at least six weeks as assessed according to RECIST 1.1 criteria and mRECIST criteria.

• Time to response (TTR): defined as the time from treatment initiation to first documented objective response (as assessed according to RECIST 1.1 criteria and mRECIST criteria).

• Duration of Response (DoR): defined as the time from the date of the first documented objective response (as assessed according to RECIST 1.1 criteria and mRECIST criteria) to the date of the first documented objective tumor progression (as assessed according to RECIST 1.1 criteria or mRECIST criteria) or the date of death from any cause, which occurs earlier. Subjects who are still alive without disease progression will be censored on the last adequate tumor assessment date. Subjects starting any subsequent anti-tumor therapy (excluding non-target bone lesions or palliative radiotherapy during treatment) and who have not previously reported progression will be censored at the time of the last tumor assessment prior to initiation of subsequent anti-tumor therapy, or at the date of initiation of subsequent anti-tumor therapy.

• Progression-free survival (PFS): defined as the time from the date of the first study treatment to the date of the first recorded tumor progression (assessed according to RECIST 1.1 or mRECIST, regardless of whether the study treatment is still ongoing) or death from any cause, which occurs earlier. Liver-specific PFS: defined as the time from treatment initiation to disease progression within the liver or death.

When determining PFS, clinical deterioration in the absence of conclusive evidence of disease progression (according to RECIST 1.1 or mRECIST) will not be considered as progression. Patients without disease progression and death will be censored at the last tumor assessment date. During the study, subjects without any tumor evaluations and death will be censored at the date of first medication. Subjects who initiate subsequent anti-tumor therapy without previously reported disease progression will be censored at the last evaluable tumor assessment date prior to initiation of subsequent anti-tumor therapy

• Overall survival (OS): defined as the time from the date of the first study treatment to death from any cause. For subjects who are still alive at the last follow-up, their OS will be censored at the time of the last date known to be alive. For subjects lose to follow-up or withdraw from study before death, their OS will be censored at the last date known to be alive

• Quality of life (QoL), which was assessed using the European Organisation for Research and Treatment of Cancer QoL questionnaire (EORTC QLQ-C30) at baseline and every six weeks until end of treatment. EORTC QLQ-C30 consists of 30 questions which assess five functional domains (physical, role, emotional, cognitive, and social), global health status/quality of life, disease/treatment related symptoms (fatigue, nausea/vomiting, pain, dyspnea, insomnia, appetite loss, constipation, and diarrhoea), and the perceived financial impact of disease.

### 7.1.2 Efficacy Evaluation Criteria

The primary and secondary efficacy endpoints of HAIC combined with camrelizumab and apatinib in the treatment of advanced hepatocellular carcinoma will be evaluated according to RECIST v1.1 or mRECIST (Annex II).

The target and non-target lesions being identified at baseline will be examined every 2 cycles (bone scan will be performed when bone progression is suspected or when CR is confirmed). It will not be affected by drug interruption or delay. If new lesions are suspected, imaging examination needs to be performed in time. The PR/CR should be confirmed after 4 weeks (28 days). If the disease progression occurs, it needs to be confirmed by imaging examination after 4 to 6 weeks (except those with rapid progression and significant clinical progression).

Survival period will be recorded and evaluated and the subjects will be followed up every 1 month for survival status by telephone, until death, loss to follow-up, withdrawal of informed consent.

Evaluation of tumor response will include all known or suspected sites of disease.

Radiological imaging studies will include computed tomography (CT) or magnetic resonance imaging (MRI) scans of the chest, abdomen, or pelvis. Brain CT or MRI will be used for subjects with known or suspected brain metastases. Bone scans will be used for patients with known or suspected bone metastases (refer to 7.1 Imaging examination for details).

If the subject’s general health deteriorates, requiring discontinuation, but there is no objective evidence of disease progression at this time, it should be reported as worsening of symptoms.

For subjects who withdrew study treatment due to reason other than disease progression (e.g. intolerable to study treatment, other conditions), an imaging examination needs to be performed at the end of the study treatment if no imaging examination is performed within 4 weeks before study treatment termination, after then, tumor assessment will be performed every 3 months until disease progression, death, or initiation of other anti-tumor therapy.

### 7.1.3 Assessment of Tumor Lesions

The first tumor imaging study at screening must have been performed within 21 days prior to enrollment. Investigators should confirm that subjects have at least one measurable lesion that meets RECIST (version 1.1) criteria. Refer to Appendix 1 for the RECIST (version 1.1) assessment methodology.

Throughout the study, assessments should be performed at the planned time points for imaging assessments specified in the protocol as far as possible. From the start of medication, the lesions found at baseline will be examined every 2 cycles ± 7 days (bone scan will be performed when bone progression is suspected or when CR is confirmed). If new lesions are suspected, they can be checked in time. The first PR/CR must be confirmed after 4 weeks ± 7 days. If the disease progresses for the first time according to RECIST (version 1.1), it needs to be confirmed by imaging examination after 4-6 weeks (except those with rapid progression and significant clinical progression). If after the first assessment of imaging progress, the subject’s clinical disease is unstable, confirmation after 4-6 weeks will not be required, and the investigator can directly determine the end the study treatment. For patients with stable disease, it should persist for a minimum of six weeks.

Unscheduled imaging evaluations can be performed at any time if patients develop clinically unstable disease during the study. Clinical disease instability is defined as follows:

(1) Clinically significant symptoms and signs (including deterioration of laboratory values) suggestive of disease progression

(2) Decreased ECOGPS score

(3) Rapid disease progression

(4) If there is tumor progression in important anatomical parts that requires other emergency medical intervention (such as spinal cord compression), if no imaging examination is performed within 4 weeks before the end of treatment, an imaging examination needs to be done when the study treatment ends or when the study is withdrawn for efficacy assessment. For patients with non-imaging evidence of progression (intolerable, other conditions), tumor assessment will be performed every 3 months until disease progression, death, or initiation of other anti-tumor therapy.

The best overall response is defined as the best response recorded during the period from the start of medication to the date of objective documentation of progression the date of initiation of subsequent anti-tumor therapy (whichever occurs first). For subjects with no documented progression or initiation of subsequent antitumor therapy, the best overall response will be determined based on all response assessments.

For subjects who continue camrelizumab after progression, the best overall response should be determined based on the response assessment recorded up to the time of initial progression. If the subject’s general health deteriorates, requiring discontinuation, but there is no objective evidence of disease progression at this time, it should be reported as worsening of symptoms.

## 7.2 Safety Analyses

### 7.2.1 Baseline Signs and Symptoms

The investigator will conduct a comprehensive physical examination of the subject during the screening period, record the subject’s vital signs and related symptoms and clinically significant abnormal results.

### 7.2.2 Physical Examination and Vital Signs

The investigator will conduct a physical examination of the subjects at each follow-up visit and record their vital signs, including respiration, heart rate, and blood pressure, according to the procedures of the study plan. After the first dose of study, new clinically significant abnormal signs on physical examination will be recorded as AEs.

The incidence and severity of adverse events (AEs) and serious adverse events (SAEs) will be judged according to the NCI-CTCAEV4.0.

Drug interruption rate and drug discontinuation rate due to adverse events.

AEs occurring during the study, including screening signs and symptoms, will be recorded in the eCRF. Drug suspension and reduction or other adjustments will be recorded in the eCRF.

### 7.2.3 Laboratory Safety Assessment

All laboratory abnormalities that are clinically significant or meet the definition of an AE/SAE should be recorded in the eCRF.

It is best for investigators to report in clinical terms rather than laboratory terms (such as anemia rather than low hemoglobin values) whenever possible.

### 7.2.4 Vital Signs and Physical Examination and Weight Measurement

Vital sign, physical examination and weight measurement will be performed according to the study protocol.

A comprehensive physical examination will be required during the screening period, and all examination results will be recorded in the eCRF.

A comprehensive physical examination will be required during the trial, and the newly occurred abnormality or deterioration in symptoms compared with baseline (screening phase) will be recorded in the eCRF. If there is no change from the screening period, there is no need to repeat recording.

## 7.3 Biomarker Analyses

The following biomarker assessments is expected in this study:

• Serum HBV DNA level and HBV surface antigen (HBsAg) negative rate during the test

• Examine tumor tissue collected at baseline and/or PD-L1 expression level in peripheral blood samples, proportion of positive cells and/or other biomarkers, such as the relationship between tumor mutational burden (TMB) at baseline and efficacy.

• Explore the relationship between alpha-fetoprotein levels and efficacy at baseline.

# 8. ADVERSE EVENT REPORT

## 8.1 Definition of Adverse Event (AE)

An adverse event is any untoward medical occurrence in a clinical investigation subject administered a pharmaceutical product, regardless of causal attribution. The trial collection will start from subjects signing informed consent and continue until at least 30 days after the last medication. Follow-up will be required to collect SAEs and irAEs within 90 days after the last dose of camrelizumab. If the subject starts new anti-tumor treatment, follow up until the start of tumor treatment (If the therapy starts within 30 days of the last medication, follow up until at least 30 days after the last medication. While if it starts more than 30 days after the last medication, follow up until the start of tumor therapy.). Adverse events can be any unfavorable undesired symptoms, signs, laboratory abnormalities or diseases, etc., including the following:

Exacerbation of pre-existing (before entering clinical trials) medical condition/disease (including exacerbation of symptoms, signs, laboratory abnormalities);

Any new adverse events: any new adverse medical condition (including symptoms, signs, newly diagnosed disease);

Abnormal laboratory values or results that are clinically significant and not due to a concomitant disease.

The researcher should record in detail any adverse events that occur to the subjects, including: the description of the adverse events and all related symptoms, the time of occurrence, the severity, the reasons for the occurrence of the adverse events, the correlation with the trial drug, the duration, the measures taken and final results and outcomes.

## 8.2 Assessment of Adverse Events

Adverse reactions are classified according to the grading standard of common toxic and side effects of anti-cancer drugs (NCICTCAE 4.0 grading standard). Adverse events that are not listed in the NCI toxicity grading criteria can be judged according to the following criteria:

Grade 1 (mild): asymptomatic or mild symptoms; clinical or diagnostic observations only; intervention not indicated;

Grade 2 (moderate): minimal, local, or non-invasive intervention indicated; limiting age-appropriate instrumental activities of daily living;

Grade 3 (severe): Severe or medically significant, but not immediately; life-threatening; hospitalization or prolongation of hospitalization indicated; disabling; or limiting self-care activities of daily living;

Grade 4: Life-threatening consequences or urgent intervention indicated;

Grade 5: Death related to adverse events.

## 8.3 Recording of Adverse Events

All adverse events, including serious adverse events, whether observed by investigators or spontaneously reported by subjects, will be collected within 30 days from the signing of the informed consent form to the last medication. Investigators will be required to fully record any adverse event, including diagnosis (if there is no diagnosis, record symptoms, signs, including laboratory abnormalities), start and end dates and times (if applicable), CTCAE severity and changes (grade 3 or above events) , whether it is a serious adverse event, the actions taken on the study drug, the treatment given due to AE and the outcome of the event, the association of adverse events with the study drug.

## 8.4 Evaluation of the Correlation Between Adverse Events and Drugs

The investigator should evaluate the possible association between the adverse events and the study drugs, according to the following criteria:

Definitely related: The time of occurrence of the response corresponds to the chronological order of the medication. Response conforms to the known type of response to the test drug. It can be improved after discontinuation and reappears with repeated medication.

Probably related: The time of occurrence of the response corresponds to the chronological order of the medication. Response conforms to the known type of response to the test drug. The patient’s clinical status or other treatment modalities may also result in this response.

Probably not related: The time of occurrence of the response does not correspond to the chronological order of the medication. Response does not fit well with the known type of response to the test drug. The patient’s clinical status or other treatment modalities may also result in this response.

Definitely not related: The time of occurrence of the response does not correspond to the chronological order of the medication. The response has a known type of response to the non-investigational drug. The patient’s clinical status or other treatment modalities may also result in this response. Response eliminates due to improvement in disease state or cessation of other treatment modalities. Response occurs with repeated use of other treatment modalities.

Unknown: There is no clear relationship between the timing of the response and the chronological order of administration. Response is similar to the known type of response to the test drug. Concomitant use of other drugs may also cause the same response.

## 8.5 Serious Adverse Event (SAE)

A serious adverse event is one of the following adverse events at any dose of the study drug, or at any time during the observation period:

·Is fatal;

·Is life threatening;

·Requires hospitalization or prolonged hospitalization;

·Results in persistent or significant disability/incapacity or substantial disruption of the patient’s ability to conduct normal life functions;

·Is a congenital anomaly/birth defect;

·Other medically important events: defined as events that endanger the subject or require medical intervention to prevent any of the above.

## 8.6 Management of Serious Adverse Events

If serious adverse events occur during the trial, the research unit must take immediate measures to protect the safety of the subjects, immediately report to the administrative department orally, and report in writing to the Safety Supervision Department of the State Drug Administration, Principal Investigator Hospital Ethics Committee within 24 hours of the occurrence of the event. The investigator should record the occurrence time, duration, measures and outcomes of serious adverse events on the report, sign and date.

Disease progression (including signs and symptoms of progression) will not be reported as SAEs, but deaths due to disease progression during the trial period are reported. The sponsor will ensure reporting procedures that meet all legal and regulatory requirements.

# 9. ADVICE ON HANDLING ADVERSE EVENTS

## 9.1 Immune-related Adverse Event

Immune-related adverse events (irAEs) are clinically significant side effects consistent with the immune mechanism of the study drug. Further serological, immunological and pathological (biopsy) data will be required to support its diagnosis. At the same time, tumor, infection, metabolism, toxin, or other pathogenic causes need to be excluded.

## 9.2 Principles of Handling Immune-related Adverse Events

The management of immune-related adverse reactions should be conducted in accordance with the medical practice and guidelines of the research institution. The following are treatment recommendations for immune-related adverse reactions.

Patients using hormones should pay attention to calcium supplementation and vitamin D3, acid suppression and gastric mucosal protection.

1. **Immune-related pneumonia**

The clinical study of camrelizumab will strengthen the monitoring of patients with signs and symptoms of immune-related pneumonia, such as cough, chest discomfort, etc. High-dose hormone therapy is used for patients with grade 2 and above after examination of chest CT. Camrelizumab can be withheld and therapy will be administered in patients with immune-related pneumonitis grade 2, and camrelizumab will be permanently discontinued in patients with grade 3 or 4. Respiratory consultation will be recommended.

Specific operations can refer to the following suggestions:

Grade 2 pneumonia: 1mg/kg/day methylprednisolone intravenously or orally at an equal dose, closely monitor CT changes, and continue to take 0.5mg/kg/day prednisone for 2 weeks after recovery to grade 1, and then reduce 5mg of prednisone every week until drug withdrawal.

Grade 3 pneumonia: 2 to 4mg/kg/day methylprednisolone intravenously or an equivalent dose intravenously, closely monitor CT changes, reduce the dose by 50% every 3 days after recovery to grade 1, 0.5mg/kg/day prednisone orally for 2 weeks, then reduce 5mg of prednisone every week until drug withdrawal.

If there are no improvement or deterioration after 3 to 5 days of hormone therapy, we will communicated with sponsor that immunosuppressive therapy can be combined.

1. **Immune-related hepatitis**

The clinical study of camrelizumab will strengthen the monitoring of patients with signs and symptoms of immune-related hepatitis, such as liver discomfort, abnormal elevation of transaminases, etc. High-dose hormone therapy will be [conduct](javascript:;)ed for patients with grade 2 and above. Specific operations can refer to the following suggestions:

Grade 2 hepatitis: 0.5 to 1mg/kg/day methylprednisolone intravenously or orally at an equal dose, closely monitor the changes in liver function indicators, and slowly reduce the dose of hormones for no less than 1 month after recovery to grade 1.

Grade 3 hepatitis: 1 to 2mg/kg/day methylprednisolone intravenously or an equivalent dose intravenously, closely monitor the changes in liver function indicators, and slowly reduce the dose of hormones for no less than 1 month after recovery to grade 1.

1. **Immune-related enteritis**

The clinical study of camrelizumab will strengthen the monitoring of the signs and symptoms of immune-related enteritis in patients, such as abdominal pain, diarrhea, hematochezia, etc. High-dose hormone therapy is [conduct](javascript:;)ed for patients with grade 2 and above. Camrelizumab can be withheld and therapy will be administered in patients with grade 2 or 3 immune-related enteritis, and camrelizumab will be permanently discontinued in patients with grade 4.

1. **Immune-related thyroid dysfunction**

Thyroid function abnormalities can occur at any time in the study, so the camrelizumab study will regularly check patients’ thyroid function status and focus on clinical symptoms of thyroid dysfunction. The patient will be treated with high-dose cortisone/prednisone after developing immune-related hyperthyroidism. Hypothyroidism will be treated with hormone replacement therapy, but glucocorticoids will not be applicable.

The clinical study of camrelizumab will strengthen the monitoring of the signs and symptoms of immune-related thyroid dysfunction in patients. High-dose hormone therapy will be [conduct](javascript:;)ed for patients with grade 3 and above hyperthyroidism, and camrelizumab will be permanently discontinued in patients with grade 4. Camrelizumab does not need to be discontinued in patients with grade ≥2 hypothyroidism.

1. **Immune-related nephritis and renal failure**

The clinical study of camrelizumab will strengthen the monitoring of the signs and symptoms of immune-related nephritis in patients. High-dose hormone therapy will be [conduct](javascript:;)ed for patients with grade 2 and above. Camrelizumab can be withheld and therapy will be administered in patients with grade 2 or 3, and camrelizumab will be permanently discontinued in patients with grade 4.

1. **Immune-related hypophysitis**

The clinical study of camrelizumab will strengthen the monitoring of the signs and symptoms of immune-related hypophysitis in patients. High-dose hormone therapy will be [conduct](javascript:;)ed for patients with grade 2 and above. Camrelizumab can be withheld and therapy will be administered in patients with grade 2 or 3, and camrelizumab will be permanently discontinued in patients with grade 4.

1. **Other immune-related adverse reactions**

In principle, according to the severity of adverse reactions, camrelizumab should be temporarily suspended. When the severity of adverse events returns to grade 1 or lower, camrelizumab can be considered again. For severe grade 3 or life-threatening grade 4 adverse reactions, camrelizumab should be permanently discontinued.

## 9.3 Infusion Reactions

Since camrelizumab is a fully humanized monoclonal antibody, it is less likely to cause infusion reactions, and no preventive medication will be required before infusion. Once the infusion reaction occurs, the infusion should be slowed down or interrupted according to the situation. Clinical supportive treatment should be given, and preventive medication should be given before subsequent medication. Acute infusion reactions (including cytokine release syndrome, angioedema, anaphylactic shock, allergic reactions, refer to NCI CTCAE v4.03 Terminology and Criteria) usually develop associated signs and symptoms during or shortly after drug infusion, and usually disappear within 24 hours after the infusion is completed. Symptoms and signs include: allergic reactions/hypersensitivity (including drug-induced fever), cough, chills, shivers, dizziness, headache, fatigue (asthenia, drowsiness), rash/peeling, pruritus of the skin/ itching, arthralgia, muscle pain, low or high blood pressure, nausea, vomiting, sweating (diaphoresis), tachycardia, tumor pain, hives (rubella), difficulty breathing (shortness of breath), or bronchospasm. Management of allergic reactions should be performed according to the medical practice and guidelines of the research institution. The following are recommendations for the treatment of infusion reactions for reference.

**Table. Treatment recommendations for infusion reactions**

| CTCAE level | Clinical symptoms | Clinical treatment | Camrelizumab therapy |
| --- | --- | --- | --- |
| Grade 1 (mild) | Mild transient reaction | Observe at the bedside and monitor closely until recovery. (Prophylaxis before infusion is recommended in the future：diphenhydramine 50mg, or equivalent and/or acetaminophen 325 to 1000mg, at least 30 minutes before camrelizumab administration) | Continue |
| Grade 3 (severe) | Moderate reaction requires treatment or drug discontinuation, which can be rapidly relieved after symptomatic treatment (such as antihistamines, non-steroidal anti-inflammatory drugs, anesthetics, bronchodilators, intravenous fluids, etc.) | Intravenous infusion of normal saline, diphenhydramine 50mg IV or equivalent and/or acetaminophen 325 to 1000mg.  Observe at the bedside and monitor closely until recovery.  Corticosteroids or bronchodilators may be considered as clinically necessary.  Original medical records record the infusion volume of the study drug.  Future prophylaxis before infusion is recommended: diphenhydramine 50mg, or equivalent and/or acetaminophen 325 to 1000mg at least 30 minutes before camrelizumab administration. Cortisol hormone (equivalent to a 25mg hydrocortisone dose) if necessary. | Discontinue. 50% of the initial infusion rate will be given when re-administered after symptoms disappear. If there are no complications within 30 minutes, the infusion rate can be increased to 100% of the original infusion rate. Monitor closely. If symptoms recur, the infusion of camrelizumab for the current treatment should be discontinued. |
| ≥ Grade 3 (severe) | Grade 3: Severe reaction without rapid remission to treatment and medication discontinuation or recurrence of symptoms after remission. Develop sequelae requiring hospitalization。Grade 4: Life-threatening. | Immediately stop the infusion of camrelizumab.  Start an intravenous infusion of normal saline.  • Recommend bronchodilator, subcutaneous injection of 1:1000 epinephrine solution 0.2 to1mg, or 0.1 to 0.25mg of 1:10000 solution of epinephrine by slow intravenous injection, if necessary, and/or diphenhydramine 50mg plus methylprednisolone 100mg or equivalent intravenous injection  • Follow the institute’s guidelines for the treatment of anaphylaxis. Observe at the bedside and monitor closely until recovery. | Permanently discontinue |

## 9.4 Symptomatic Management of Apatinib-related Adverse Reactions

(1) Hand-foot syndrome (HFS)

Hand-foot syndrome, palm-plantar dysesthesia or acral erythema, is a cutaneous toxicity that will be more pronounced in areas of compression or stress. Cancer patients can appear during chemotherapy or molecularly targeted therapy. HFSR is characterized by numbness, hypoesthesia, paresthesia, tingling, painlessness or pain, skin swelling, or erythema, desquamation, chapping, induration-like blisters, and severe pain etc.

HFS classification:

Grade 1: Numbness/dysesthesia/paresthesia of the hands and/or feet, painless swelling or erythema and/or discomfort that does not interfere with normal activities.

Grade 2: Painful erythema and swelling of the hands and/or feet and/or discomfort that influences the patient’s daily activities

Grade 3: Wet desquamation, ulceration, blisters, or severe pain of the hands and/or feet and/or severe discomfort that prevents the patient from working or performing daily activities. Intense pain and loss of skin function are relatively rare.

Symptomatic treatment and management of HFS: Take some necessary symptomatic and supportive treatment, including: strengthening skin care, keeping skin clean, and avoiding secondary infection. Avoid pressure or friction. Use a moisturizer or lubricant and topically use lotion or lubricant containing urea and corticosteroids. Topically give anti-fungal or antibiotic treatment if necessary.

Note: If the hand-foot syndrome of grade 3 or above occurs 3 times in a row, and there is a trend of aggravation, the medication will be terminated and the clinical study will be withdrawn.

(2) Hypertension

Before enrolling patients, the blood pressure requirements in the inclusion and exclusion criteria should be strictly followed. Hypertensive patients can complete blood pressure control by adjusting the dose of anti-hypertensive drugs or adding new anti-hypertensive drugs before taking the study drug. Before randomization, the blood pressure must be controlled within 140/90mmHg (Interval of 24 hours or more, 2 blood pressure monitoring average).

Monitoring and management of this type of hypertension: Blood pressure monitoring should be performed at least 3 times a week during the first 2 cycles of targeted drug therapy

Since anti-VEGF/VEGFR targeted therapies cause a decrease in NO synthesis and eventually activate the renin-angiotensin-aldosterone system to cause hypertension, angiotensin-converting enzyme (ACE) inhibitors (such as captopril, enalapril, benazepril, silapril, etc.) are the best choice for antihypertensive therapy. Some patients who are allergic or intolerant to ACE inhibitors can be treated with angiotensin II receptor blockers (ARB, such as losartan, valsartan, irbesartan and telmisartan, etc.). In addition to lowering blood pressure, ARB are also beneficial for relieving proteinuria. ACE inhibitor can be used for patients with chronic kidney disease, proteinuria and metabolic syndrome. Dihydropyridine calcium antagonists are suitable for elderly patients.

Patients with hypertension or aggravation of hypertension during medication should: (1) Adjust the research substance according to the protocol (refer to the table below); (2) Start taking anti-hypertensive drugs or adjust the dose of anti-hypertensive drugs.

In the trial, it is recommended to choose drugs for the treatment of hypertension: (1) angiotensin-converting enzyme inhibitor (ACEI); (2) angiotensin II receptor antagonist (ARB); (3) dihydropyridine calcium channel antagonist; (4) β-receptor blocker.

The use of diuretic antihypertensive drugs will not be recommended, and anti-hypertensive drugs with CYP3A4 inhibitory effects such as nicardipine, diltiazem, and verapamil will be forbidden while taking the experimental drugs. For patients with hypertensive crisis, apatinib should be discontinued.

(3) Bleeding

Gastrointestinal bleeding, including fecal occult blood (++) and above, hematemesis or bloody stool, should be treated symptomatic actively. Patients with upper gastrointestinal bleeding should be fasted, and given antacids, gastric mucosa protection, hemostasis (transamin, reptilase, etc.). Octreotide can be used if necessary. Patients with lower gastrointestinal bleeding should be given hemostasis, blood transfusion and supportive treatment. Patients with uncontrolled bleeding should seek surgical assistance immediately.

Patients with hemoptysis should be given hemostasis, blood transfusion and supportive treatment. Patients with uncontrolled bleeding should seek surgical assistance.

Note: For patients diagnosed with cerebral hemorrhage, pulmonary hemorrhage of grade II or above, or hemorrhage of grade III or above, the drug must be discontinued immediately. The apatinib administration should be discontinued and the administration of camrelizumab should be suspended. After the symptoms are relieved and disappear, consider whether to continue camrelizumab as appropriate.

(4) Proteinuria

During the entire treatment period, closely monitor proteinuria in all patients, and strengthen monitoring those with a history of hypertension. For 2 consecutive urine protein ++ to +++, 24-hour urine protein measurement will be required. For urine protein ++++ and above, 24-hour urine protein measurement will be required.

Note: If nephrotic syndrome occurs, the drug should be permanently discontinued and the patients will withdraw from this study.

(5) Thrombosis

If any arterial thrombosis (such as cerebral ischemia, stroke, angina pectoris, myocardial infarction, etc.) occurs, the drug should be stopped immediately and the patients will withdraw from the study. If symptomatic IV venous thrombosis occurs, the drug should be discontinued and the patients will withdraw from the study. Symptoms of thrombosis should be treated immediately with symptomatic treatment, surgery, or anticoagulant medication, etc.

(6) Fatigue, weakness

Fatigue and weakness are common clinical symptoms related to tumors. Electrolyte imbalance, abnormal liver function, abnormal heart function, etc. all may cause fatigue and weakness. At the same time, fatigue and weakness are also common clinical adverse reactions of neovascular targeting drugs such as sunitinib, pazopanib, and sorafenib. Clinical reports show that neovascular targeting drugs may increase the incidence of fatigue and weakness by causing hypothyroidism.

In previously completed apatinib clinical trials, the incidence of fatigue and weakness in the apatinib trial group was higher than that in the control group. The specific mechanism of apatinib-induced increase in the incidence of fatigue and weakness remains unclear.

Therefore, when a patient develops and reports fatigue and weakness of grade II and above, it should be taken seriously. When fatigue and weakness of grade III and above occur, the patient should be immediately admitted to the hospital for examination, and checked one by one to exclude electrolyte disturbance, abnormal liver function, and cardiac dysfunction (ECG, cardiac ultrasound), abnormal hormone levels (adrenal hormones, thyroid hormones) and other possible causes and symptomatic treatment should be given. Suspend or adjust dose of the test drug according to the principle of dose adjustment.

(7) Abdominal pain

Abdominal pain is not uncommon during apatinib treatment and is mostly associated with tumor symptoms. At the same time, gastrointestinal perforation has occasionally occurred in clinical trials of apatinib and other types of anti-neovascular drugs. For patients with abdominal pain symptoms, researchers should be alert to the possibility of gastrointestinal perforation. Once gastrointestinal perforation is found, the drug should be discontinued immediately. The patients should withdraw from the study, and active symptomatic treatment should be taken.

(8) Pulmonary interstitial fibrosis

Clinicians should fully know about the patient’s disease and be familiar with drugs that may cause pulmonary toxicity. The clinical symptoms and X-ray or CT changes of the patients should be closely observed. Once patients develop symptoms such as unexplained cough, chest tightness, suffocation, dyspnea, and hemoptysis, the cause should be identified in time, and the drug should be discontinued as soon as other causes (such as infection and heart failure) are excluded. Bronchoalveolar lavage examination and surgical lung biopsy are important means of diagnosing interstitial lung disease. At present, there is no satisfactory treatment plan for pulmonary fibrosis. It is recommended to refer to the guidelines for the diagnosis and treatment of idiopathic pulmonary (interstitial) fibrosis issued by the Respiratory Branch of the Chinese Medical Association, to correct hypoxemia and use glucocorticoids and other drugs in time.

# 10.STATIATICAL ANALYSES

## 10.1 Sample Size Determination

This is a prospective exploratory study which intends to adopt the Simon two-stage study design. According to previous studies, the expected effective rate of the new treatment could achieve 60.8%, and the poor effective rate is set at 40%. To control type II error rate no more than 0.20 at a one-sided significance level of 0.025 and, the two-stage design requires 26 patients to be enrolled in the first stage, the study treatment will be considered non-effective and study will be terminated if no more than 11 patients (< 12 patients) achieve CR/PR, otherwise another 21 patients will be enrolled. The study treatment will be considered with promising effectiveness if more than 25 patients in total achieve CR/PR.

## 10.2 Analysis Set

• Full Analysis Set (FAS): According to the ITT principle, it consists of all enrolled subjects who receive at least one time of study treatment. FAS is the primary analysis set for demographic and baseline characteristics and efficacy analysis.

• Safety Analysis Set (SS): Consists of all subjects who receive at least one time of the study treatment.

• Per Protocol Set (PPS): A subset of FAS, consisting of subjects who do not experience major protocol deviation which are likely to affect the efficacy. Protocol deviations which lead subjects to be excluded from PPS will be determined before database lock. PPS will be used for sensitivity analysis of the primary efficacyendpoints and key secondary efficacy endpoints.

• Patients reported outcome analysis set (PRO): A subset of FAS, consisting of subjects who received at least one time of study treatment, with baseline EORTC QLQ-C30 data available and with at least one post-baseline EORTC QLQ-C30 questionnaire being answered.

## 10.3 Endpoint Analysis and Statistical Methods

### 10.3.1 General Methods of Statistical Analysis

All statistical analysis will be performed by using SAS9.4 (or higher). Unless otherwise specified, all the confidence intervals (CIs) are reported at 2-sided 95% confidence level.

Unless otherwise specified, quantitative variable will be summarized using descriptive statistics, including number of subjects (n), mean, median, standard deviation (Std), minimum and maximum. Qualitative variables will be summarized by count(s) and percentages (%) and the calculation of percentages will be based on the total number of subjects in the relevant analysis set.

### 10.3.2 Efficacy Analyses

#### 10.3.2.1 Primary Endpoint Analysis

ORR: The proportion of subjects in the analysis set who reached complete response (CR) or partial response (PR) according to RECIST (version 1.1) criteria (formula below). The primary analysis of ORR will be performed in FAS. ORR and its corresponding exact 95% CI (Clopper-pearson method) will be provided for FAS and subgroups of interest. In addition, the number and percentage of subjects whose best overall response are CR, PR, stable disease (SD) and progressive disease (PD) will also be presented.

#### 10.3.2.2 Secondary Endpoint Analyses

PFS: defined as the time from the date of the first study treatment to the date of the first recorded tumor progression (assessed according to RECIST 1.1 or mRECIST) or death of any cause, whichever occurs earlier..

When determining PFS, clinical deterioration in the absence of conclusive evidence of disease progression (according to RECIST 1.1 or mRECIST) will not be considered as progression. Only the data collected before the initiation of subsequent anti-tumor therapy will be used for PFS analysis. Subjects who receive subsequent anti-tumor therapy before PD/death will be censored at last adequate tumor assessment before the initiation of subsequent anti-tumor therapy. Subjects who missed two or more consecutive tumor assessment immediate before PD/death will be censored on the date of last adequate tumor assessment before the consecutive missing of the tumor assessments. Subjects who are still alive without disease progression and without the initiation of subsequent anti-tumor therapy will be censored at last adequate tumor assessment. PFS will be censored at the time of first study treatment administration in case there is no post-baseline tumor assessment unless early death occurs.

The number and percentage of PFS events and the subjects being censored will be summarized. Furthermore, the type of events and reasons for censor will also be summarized. The 6- and 12-month disease progression-free rates the median PFS as well as their corresponding 95% CI will be estimated using Kaplan Meier method. In addition, the Kaplan-Meier curve of PFS will also be provided.

OS is defined as the time from the date of the first study treatment to the death from any cause. Subjects who are still alive at the date of data cut-off will be censored at the last date known to be alive. Subjects lose to follow-up or withdraw from study before death will also be censored at the last date known to be alive.

Similar analysis methods of PFS will be performed for OS. The number and percentage of death and subjects being censored will be summarized, the reason of censor will also be summarized. The 6-/9-/12-month overall survival rate and median OS as well as their corresponding 95% CI will be estimated by using Kaplan-Meier method, and the Kaplan-Meier curve will also be presented.

DCR is defined as the proportion of subjects in the analysis set whose BOR achieve CR, PR or SD according to RECIST 1.1 or mRECIST criteria. DCR and its corresponding exact 95% CI (by clopper-pearson method) will be presented.

Time to response (TTR) was defined as the time from treatment initiation to first documented response (as assessed according to RECIST 1.1 criteria and mRECIST criteria).

DOR is defined as the time from the first time the subjects meets the CR/PR criteria defined by RECIST 1.1to the first occurrence of disease progression (as defined by RECIST 1.1 or mRECIST) or death from any cause, which occurs earlier. Subjects who are still alive without disease progression at the end of study will be censored on the last tumor assessment date. The censor rules of DoR is the same as those of PFS.

The Kaplan-Meier method will be used to estimate the median duration of response (DOR) and its 95% CI. The Kaplan-Meier curve of DoR will be presented.

### 10.3.3 Safety Analysis

The safety analysis will be performed in SS. The safety and tolerability of the drug will be evaluated from the aspects of AEs, death, laboratory data, vital signs, etc.

#### 10.3.3.1 Exposure of Medication

The exposure to the study treatment during the study period will be summarized, including the total exposure duration, number of dose adjustment, the total dose, dose intensity and relative dose intensity of each study drug will be summarized.

#### 10.3.3.2 Adverse Events

Treatment-emergent adverse events (TEAEs), is defined as adverse events occur or become severe on or after the date of first study treatment administration. The incidence of TEAEs， TEAEs, serious TEAEs (SAEs) , immune-related adverse events (irAEs), TEAEs leading to discontinuation of the study drug, TEAEs leading to death will be summarized. The TEAEs mentioned above will further be analyzed by their relationship to the study treatment and by severity (especially for grade 3 or above).

### 10.3.4 Baseline Characteristics of Subjects

Demographic characteristics and other baseline information listed below will be summarized by descriptive statistics for FAS.

- Age as continuous variable
- Age (≥ 50 vs. < 50)
- Gender (Male, female)
- Body weight index (BMI) as continuous variable
- BMI (≥24 kg/m2, < 24 kg/m2)
- Etiology
- ECOG performance score (0, 1, 2)
- BCLC stage
- Child-Pugh score (5, 6)
- ALBI grade (Grade 1, Grade 2)
- AFP level (ng/mL) (≥ 400 ng/mL, < 400 ng/mL)
- PIVKA-II (mAU/mL) as continuous variable
- PIVKA-II (mAU/mL) (≥ 10000 mAU/mL, < 10000 mAU/mL)
- Tumor size (cm) (≥ 10 cm, < 10cm)
- Tumor number (Single, Multiple)
- Venous Tumor Thrombus (Present, Absent)
- Portal Vein Tumor Thrombus (vp1, vp2, vp3, vp4, Absent)
- Extrahepatic Metastasis (Present, Absent)

### 10.3.5 Exploratory Analysis

Descriptive statistics will be performed on the baseline PD-L1 expression of the overall subjects, and the proportion of subjects with different PD-L1 expression levels and the relationship with the efficacy will be analyzed.

EORTC QLQ-C30 EORTC QLQ-C30 consists of 30 questions which assess five functional domains (physical, role, emotional, cognitive, and social), global health status/quality of life, disease/treatment related symptoms (fatigue, nausea/vomiting, pain, dyspnea, insomnia, appetite loss, constipation, and diarrhoea), and the perceived financial impact of disease.

The subscales of EORTC QLQ-C30 will be scored based on the EORTC scoring manual. Each scale of the EORTC QLQ-C30 will be transformed to a 0 to 100 range. After transforming, higher score represents “better” condition for functional scales and “worse” condition for symptom scales/items.

The transformed scale scores of each scale will be summarized for baseline and each post-baseline scheduled visit by using descriptive statistics.

The score of a scale at certain timepoint will be treated as missing if less than half of the items within the scale are not answered at the corresponding timepoint. Deterioration is defined as decrease from baseline for at least 10 points and maintained for two consecutive timepoints, or one timepoint followed by death from any cause within 140 days for the selected functional scales. Similarly, deterioration is defined as increase from baseline for at least 10 points and maintained for two consecutive timepoints, or one timepoint followed by death from any cause within 140 days for the selected symptom scales.

Time to deterioration (TTD) is defined as the time form first study treatment administration to the first time of deterioration criteria are met.

The median TTD for each scale and the corresponding 95% CI will be estimated by Kaplan-Meier method. The Kaplan Meier curves will also be presented.

# 11.DATA COLLECTION AND MANAGEMENT

According to the guidelines of GCP, investigators should follow the corresponding standard operating procedures to ensure that the conduct of clinical trials and the collection, recording and reporting of data comply with the protocol, GCP and corresponding regulatory requirements.

## 11.1 Case Report Form

The main purpose is to obtain the necessary information for the research protocol in a complete, accurate, clear and timely manner. The data in the case report form should be consistent with the original document.

The filling of the case report form must be complete and clear (use a black or blue ballpoint pen and be in accordance with legal document requirements). All revisions and corrections must be made and confirmed by the investigator, with the date of revision/correction noted. Errors must be kept clearly and cannot be covered by correction data (such as using correction fluid). The researcher must indicate his/her reasons for revising important data.

For missing information/notes in the medical record, the blank input in the medical record report form should be replaced by underlined cancellation to avoid unnecessary follow-up investigation.

The case report form is a regulatory document and must be suitable for submission to hospital authorities.

## 11.2 Data Management

In compliance with ICH/GCP guidelines, the investigator/institution will maintain the CRF and all source documents supporting the collection of data from each subject, all study documentation specified in the Clinical Study Conduct Important Documents, and all study documentation specified in applicable regulatory requirements. The investigator/institution will take measures to prevent accidental or premature destruction of these documents. Important documentation must be retained until at least 2 years after the official discontinuation of clinical development of the study drug.

# 12.Ethics

## 12.1 Responsibility of the Investigator

Investigators should be responsible for ensuring that clinical research is carried out according to the research protocol, and the ethical and moral principles are based on the Declaration of Helsinki (Declaration of the World Medical Association of Helsinki, the current revision) and Good Clinical Practice (GCP), with appropriate adjustments according to China’s national conditions. The above documents must be stated on the subject’s informed consent form, which is a basic prerequisite for inclusion in clinical research.

## 12.2 Ethics Committee

Before the start of the study, the study protocol and related documents (patient information, informed consent, investigator manual, etc.) should be submitted to the ethics committee, and their consent should be obtained. study documents with ethics committee approval should be archived. Study can only be conducted with written consent.

Any revision to the study protocol should be referred to the ethics committee and serious adverse events should be reported to the IRB in accordance with international and/or local requirements.

## 12.3 Information of Subjects

Signed written informed consent is a prerequisite for patient enrollment. Before signing, the investigator must provide the subject with sufficient information. If it complies with hospital regulations, information can be provided to the subjects through the executive staff designated by the investigator. In addition to written information, the investigator or executive staff should inform the patient orally. Pay attention to the choice of wording so that the subject can fully and easily understand it.

The subject information form should be revised whenever important new information is discovered and the patient’s informed consent is involved.

## 12.4 Informed Consent

Written consent from patients willing to participate in clinical study will be required prior to all research-related activities. The informed consent form needs to be signed by the patient and the investigator/investigator-designated executive staff, and the date of signature should be marked.

The investigator should reconfirm whether to sign the consent form in the CRF. The signed and dated informed consent form should be deposited with the investigator and must be securely archived by the investigator for monitoring, review, and inspection at any time. The original copy of the informed consent form should be provided to the patient before conducting the study.

If the patient or legally recognized representative cannot read the informed consent, a reliable and independent witness should be present throughout the discussion of the informed consent form. The choice of witnesses must guarantee the patient's right to confidentiality. A reliable and independent witness should be an independent individual, not a person affiliated with the research institution or participating in the study. Family members or acquaintances are suitable candidates for independent witnesses. If possible, after the subject or patient or legally recognized representative has given verbal consent and signed the informed consent form, the witness should personally sign and date the informed consent form to certify that the information is accurate. Because only the patient or legally recognized representative fully understands the content of the informed consent, it is a real informed consent.

# REFERENCES:

1. Bray F, Ferlay J, Soerjomataram I, Siegel RL, Torre LA, Jemal A. Global cancer statistics 2018: GLOBOCAN estimates of incidence and mortality worldwide for 36 cancers in 185 countries. CA: a cancer journal for clinicians. 2018;68(6):394-424.

2. Villanueva A. Hepatocellular Carcinoma. The New England journal of medicine. 2019;380(15):1450-62.

3. Forner A, Reig M, Bruix J. Hepatocellular carcinoma. Lancet (London, England). 2018;391(10127):1301-14.

4. Bruix J, Raoul JL, Sherman M, Mazzaferro V, Bolondi L, Craxi A, et al. Efficacy and safety of sorafenib in patients with advanced hepatocellular carcinoma: subanalyses of a phase III trial. Journal of hepatology. 2012;57(4):821-9.

5. Kudo M, Finn RS, Qin S, Han KH, Ikeda K, Piscaglia F, et al. Lenvatinib versus sorafenib in first-line treatment of patients with unresectable hepatocellular carcinoma: a randomised phase 3 non-inferiority trial. Lancet (London, England). 2018;391(10126):1163-73.

6. Bruix J, Qin S, Merle P, Granito A, Huang YH, Bodoky G, et al. Regorafenib for patients with hepatocellular carcinoma who progressed on sorafenib treatment (RESORCE): a randomised, double-blind, placebo-controlled, phase 3 trial. Lancet (London, England). 2017;389(10064):56-66.

7. Tian S, Quan H, Xie C, Guo H, Lu F, Xu Y, et al. YN968D1 is a novel and selective inhibitor of vascular endothelial growth factor receptor-2 tyrosine kinase with potent activity in vitro and in vivo. Cancer science. 2011;102(7):1374-80.

8. Li J, Zhao X, Chen L, Guo H, Lv F, Jia K, et al. Safety and pharmacokinetics of novel selective vascular endothelial growth factor receptor-2 inhibitor YN968D1 in patients with advanced malignancies. BMC cancer. 2010;10:529.

9. Xue JM, Astere M, Zhong MX, Lin H, Shen J, Zhu YX. Efficacy and safety of apatinib treatment for gastric cancer, hepatocellular carcinoma and non-small cell lung cancer: a meta-analysis. OncoTargets and therapy. 2018;11:6119-28.

10. Schildberg FA, Sharpe AH, Turley SJ. Hepatic immune regulation by stromal cells. Current opinion in immunology. 2015;32:1-6.

11. Miroux C, Vausselin T, Delhem N. Regulatory T cells in HBV and HCV liver diseases: implication of regulatory T lymphocytes in the control of immune response. Expert opinion on biological therapy. 2010;10(11):1563-72.

12. Lin CL, Kao JH. Risk stratification for hepatitis B virus related hepatocellular carcinoma. Journal of gastroenterology and hepatology. 2013;28(1):10-7.

13. Wu H, Chen P, Liao R, Li YW, Yi Y, Wang JX, et al. Intratumoral regulatory T cells with higher prevalence and more suppressive activity in hepatocellular carcinoma patients. Journal of gastroenterology and hepatology. 2013;28(9):1555-64.

14. Kobayashi N, Hiraoka N, Yamagami W, Ojima H, Kanai Y, Kosuge T, et al. FOXP3+ regulatory T cells affect the development and progression of hepatocarcinogenesis. Clinical cancer research : an official journal of the American Association for Cancer Research. 2007;13(3):902-11.

15. Shen P, Wang A, He M, Wang Q, Zheng S. Increased circulating Lin(-/low) CD33(+) HLA-DR(-) myeloid-derived suppressor cells in hepatocellular carcinoma patients. Hepatology research : the official journal of the Japan Society of Hepatology. 2014;44(6):639-50.

16. Schrader J. The role of MDSCs in hepatocellular carcinoma--in vivo veritas? Journal of hepatology. 2013;59(5):921-3.

17. Desbois M, Champiat S, Chaput N. [Breaking immune tolerance in cancer]. Bulletin du cancer. 2015;102(1):34-52.

18. Murakami N, Riella LV. Co-inhibitory pathways and their importance in immune regulation. Transplantation. 2014;98(1):3-14.

19. El-Khoueiry AB, Sangro B, Yau T, Crocenzi TS, Kudo M, Hsu C, et al. Nivolumab in patients with advanced hepatocellular carcinoma (CheckMate 040): an open-label, non-comparative, phase 1/2 dose escalation and expansion trial. Lancet (London, England). 2017;389(10088):2492-502.

20. Zhu AX, Finn RS, Edeline J, Cattan S, Ogasawara S, Palmer D, et al. Pembrolizumab in patients with advanced hepatocellular carcinoma previously treated with sorafenib (KEYNOTE-224): a non-randomised, open-label phase 2 trial. The Lancet Oncology. 2018;19(7):940-52.

21. Lyu N, Lin Y, Kong Y, Zhang Z, Liu L, Zheng L, et al. FOXAI: a phase II trial evaluating the efficacy and safety of hepatic arterial infusion of oxaliplatin plus fluorouracil/leucovorin for advanced hepatocellular carcinoma. Gut. 2018;67(2):395-6.

22. Lyu N, Kong Y, Mu L, Lin Y, Li J, Liu Y, et al. Hepatic arterial infusion of oxaliplatin plus fluorouracil/leucovorin vs. sorafenib for advanced hepatocellular carcinoma. Journal of hepatology. 2018;69(1):60-9.

23. He M, Li Q, Zou R, Shen J, Fang W, Tan G, et al. Sorafenib Plus Hepatic Arterial Infusion of Oxaliplatin, Fluorouracil, and Leucovorin vs Sorafenib Alone for Hepatocellular Carcinoma With Portal Vein Invasion: A Randomized Clinical Trial. JAMA oncology. 2019.

24. Emens LA, Middleton G. The interplay of immunotherapy and chemotherapy: harnessing potential synergies. Cancer immunology research. 2015;3(5):436-43.

25. Xu J, Zhang Y, Jia R, Yue C, Chang L, Liu R, et al. Anti-PD-1 Antibody 卡瑞利珠单抗 Combined with Apatinib for Advanced Hepatocellular Carcinoma, Gastric, or Esophagogastric Junction Cancer: An Open-label, Dose Escalation and Expansion Study. Clinical cancer research : an official journal of the American Association for Cancer Research. 2019;25(2):515-23.

26. Zhao S, Ren S, Jiang T, Zhu B, Li X, Zhao C, et al. Low-Dose Apatinib Optimizes Tumor Microenvironment and Potentiates Antitumor Effect of PD-1/PD-L1 Blockade in Lung Cancer. Cancer immunology research. 2019;7(4):630-43.

27. Huang Y, Kim BYS, Chan CK, Hahn SM, Weissman IL, Jiang W. Improving immune-vascular crosstalk for cancer immunotherapy. Nature reviews Immunology. 2018;18(3):195-203.

28. Khan KA, Kerbel RS. Improving immunotherapy outcomes with anti-angiogenic treatments and vice versa. Nature reviews Clinical oncology. 2018;15(5):310-24.

29. Jain RK. Antiangiogenesis strategies revisited: from starving tumors to alleviating hypoxia. Cancer cell. 2014;26(5):605-22.
